# A comparative study of *CDO1* promoter methylation in gastric cancer and *H. pylori*-associated chronic gastritis: Implications for diagnostic performance

**DOI:** 10.1371/journal.pone.0335832

**Published:** 2026-08-12

**Authors:** Selin Kankaya, Metehan Karatas, Engin Hatipoglu, Nuray Kepil, Zulal Kaptan, Zeynep Caliskan, Matem Tuncdemir, Yildiz Dincer

**Affiliations:** 1 Department of Medical Biochemistry, Faculty of Medicine, Istanbul Yeni Yuzyil University, Istanbul, Turkey; 2 Department of Medical Biology, Faculty of Medicine, Istanbul University-Cerrahpasa, Istanbul, Turkey; 3 Department of Surgical Medical Sciences, Faculty of Medicine, Istanbul University-Cerrahpasa, Istanbul, Turkey; 4 Department of Pathology, Faculty of Medicine, Istanbul University-Cerrahpasa, Istanbul, Turkey; 5 Department of Physiology, Faculty of Medicine, Bezmialem Vakif University, Istanbul, Turkey; 6 Department of Medical Biochemistry, Faculty of Medicine, Istanbul University-Cerrahpasa, Istanbul, Turkey; Nelson Mandela African Institute of Science and Technology, TANZANIA, UNITED REPUBLIC OF

## Abstract

Silencing of the *Cysteine dioxygenase-1 (CDO1)* tumor suppressor gene by aberrant DNA methylation contributes to gastric carcinogenesis. Despite evidence that *Helicobacter pylori (H. pylori)* infection induces aberrant DNA methylation in the gastric mucosa, *CDO1* promoter methylation has not been well characterized in *H. Pylori* positive (HPP) patients with chronic gastritis. This study aimed to investigate the *CDO1* promoter methylation levels in patients with chronic gastritis with and without *H. pylori* infection, in comparison with gastric tumors. The quantitative analysis of *CDO1* promoter methylation and *CDO1* immunopositivity was performed in 45 primary gastric tumors, 17 biopsy samples of HPP chronic gastritis, 23 *H. Pylori* negative (HPN) chronic gastritis, and 15 normal gastric mucosa samples (control group). *CDO1* expression was evaluated by immunohistochemistry, and promoter methylation levels were determined using quantitative methylation-specific PCR following bisulfite conversion. *CDO1* promoter methylation levels were significantly higher in the gastric cancer (GC) group compared to the HPN chronic gastritis and control groups (p < 0.001). No significant difference was observed between the GC and HPP chronic gastritis groups. However, *CDO1* promoter methylation levels were significantly higher in the HPP group compared to the HPN group (p = 0.049). No significant differences in *CDO1* immunopositivity were observed among the study groups. ROC analysis demonstrated good discriminative ability of *CDO1* methylation between GC and non-cancer cases (AUC = 0.83), whereas its performance was more limited in distinguishing GC from HPP chronic gastritis (AUC = 0.67). *CDO1* promoter methylation is increased in GC and HPP chronic gastritis, suggesting that *H. pylori*-associated chronic inflammation may be associated with early epigenetic alterations. Although no direct correlation with protein expression was observed, our findings suggest that while *CDO1* methylation may be informative for identifying cancer-related epigenetic changes, it may be insufficient as a standalone biomarker in high-risk inflammatory conditions. Further studies are needed to clarify its clinical utility, particularly in high-risk populations.

## Introduction

Gastric cancer is the fifth most common malignancy worldwide and remains a leading cause of cancer-related mortality, according to Global Cancer Statistics (GLOBOCAN) 2022 [1]. The presence of precancerous conditions, particularly *Helicobacter pylori* (*H. pylori*) infection, is a well-established risk factor for gastric carcinogenesis. Although endoscopic examination plays a crucial role in the early detection of gastric cancer, its invasive nature and limited patient compliance restrict its widespread use in screening and follow-up. Therefore, there is a growing need for non-invasive approaches that can be utilized for early diagnosis, risk stratification, and disease monitoring, as well as for identifying potential molecular biomarkers and therapeutic targets.

Chronic gastritis, particularly that associated with *H. pylori* infection, is a critical premalignant stage in the multistep cascade leading to gastric cancer (GC) [2]. Genetic and epigenetic alterations play a crucial role in the development of GC. Among the key epigenetic mechanisms, DNA methylation is particularly significant in regulating gene expression. This process involves the addition of a methyl group to the carbon-5 position of the cytosine ring preceding a guanine nucleotide (CpG dinucleotide), resulting in the formation of 5-methylcytosine (5-CH_3_-cytosine), and is catalyzed by *DNA methyltransferases* (DNMTs). In gastric cancers, aberrant DNA methylation events are observed more frequently than mutations and other genetic abnormalities, highlighting the pivotal role of epigenetic modifications in the development and progression of the disease [3]. Since DNA methylation is a reversible epigenetic modification, targeting and restoring these abnormal methylation patterns may represent a promising therapeutic strategy in cancer management.

Chronic inflammation induced by *H. pylori* infection promotes aberrant DNA methylation in gastric mucosal cells. Notably, hypermethylation of promoter CpG islands in tumor suppressor genes such as Secreted Frizzled-Related Protein 1 (*SFRP1)* [4], Cyclin-Dependent Kinase Inhibitor 2A (*CDKN2A)* [5], Cadherin 1 (*CDH1)* [6], and *Cysteine dioxygenase 1* (*CDO1)* [7] leads to their transcriptional silencing, thereby contributing to the development of GC.

Similarly, epigenetic silencing of the mentioned tumor suppressor genes results from dysregulation of methylation-related genes due to exposure to cytokines produced by innate immunity [8–10]. The *Ten-eleven translocation* (*TET)* genes are involved in DNA demethylation. Activation of the Nuclear Factor Kappa B (*NF-κB)* pathway causes an increase in expression of *TET*-targeting miRNAs, which in turn down-regulates *TET* expression. In addition, the activities of DNMTs are increased by exposure to nitric oxide (NO) [9]. *NF-κB* activation and increased NO generation are consistently observed in *H. pylori-*induced gastritis [11].

*CDO1* is a tumor suppressor gene. It encodes a non-heme, iron-dependent enzyme that regulates redox homeostasis through glutathione and promotes cell death under oxidative stress [12]. *CDO1* expression is epigenetically suppressed in tumor cells through promoter hypermethylation, which contributes to the evasion of apoptosis [13].

Hypermethylation in the promoter region of *CDO1* has been reported determined in GC [7], but the methylation status of *CDO1* has not been examined in chronic gastritis to date. Given that gastric carcinoma often arises in the context of chronic gastritis that *H. pylori* positivity is frequently detected in patients with chronic gastritis, we hypothesized that aberrant methylation in the promoter region of *CDO1* may be a possible link between chronic gastritis and GC. Previous studies have primarily evaluated *CDO1* methylation by comparing gastric cancer tissues with normal controls, with limited data addressing its role in clinically relevant precursor conditions such as *H. pylori*-associated chronic gastritis [7,14].

In the present study, we examined the methylation level of the *CDO1* promoter region and the *CDO1* immunopositivity in primary gastric tumors and biopsy samples of chronic gastritis, with and without *H. pylori* infection. In addition, we aimed to assess the discriminative performance of *CDO1* methylation in distinguishing gastric cancer from both non-cancer conditions and *H. pylori*-associated chronic gastritis, which represents a clinically relevant high-risk group.

## Materials and methods

### Study design and clinical samples

This study was conducted in accordance with the Declaration of Helsinki, and the protocol used was approved by the Clinical Research Ethics Committee of Cerrahpasa Medical Faculty (07/2020-83045809-604.01.02). All tissue samples were obtained from patients treated at Cerrahpasa Medical Faculty Hospital between 10/11/2010 and 01/03/2020. Their data were retrospectively accessed for research purposes between 10/02/2022 and 10/03/2022. All participants provided written informed consent prior to inclusion in the study.

The required sample size was calculated using G*Power 3.1.9.6 software. A minimum of 15 participants per study group was determined based on a one-way ANOVA, assuming a large effect size (f = 0.40), a significance level (α) of 0.05, and a power (1 – β) of 0.90. Accordingly, four study groups were formed, each consisting of at least 15 cases: Control group (n = 15), *H. pylori* negative (HPN) chronic gastritis group (n = 23), *H. pylori* positive (HPP) chronic gastritis group (n = 17), and GC group (n = 45).

The chronic gastritis groups, which include patients with and without *H. pylori* infection, consists of individuals who underwent upper gastrointestinal endoscopic examination and were diagnosed with chronic gastritis based on the histopathological evaluation of biopsy samples. *H. pylori* infection status in chronic gastritis patients was determined by histopathological examination of gastric biopsy specimens using modified Giemsa staining, a widely accepted routine diagnostic method in clinical pathology practice. None of the chronic gastric patients had a malignant neoplasm. The control group is consisted of normal mucosa samples obtained from individuals without *H. pylori* infection.

The GC group consisted of patients diagnosed with primary gastric adenocarcinoma who underwent curative gastrectomy at Cerrahpasa Medical Faculty Hospital. Cases who had received chemotherapy or radiotherapy prior to surgery were excluded from the study. Formalin-fixed, paraffin-embedded (FFPE) samples obtained from the antrum, archived in the pathology department, were used for methylation and immunohistochemical analysis.

Tumor purity, defined as the proportion of cancer cells within the tumor tissue, was assessed by microscopic evaluation performed by an experienced pathologist. Only tissue sections containing at least 30% tumor cells, as assessed by an experienced pathologist, were included in the analysis to ensure sufficient tumor DNA for reliable PCR-based methylation analysis, in line with commonly adopted practices in molecular pathology [15,16]. The clinical classification of gastric carcinoma was performed according to the 2017 American Joint Committee on Cancer (AJCC) staging system [17].

### Analysis of the *CDO1* gene promoter DNA methylation

The percentage of DNA methylation levels of the *CDO1* gene in primary gastric tumors and biopsy samples was determined using quantitative methylation-specific PCR (Q-MSP) following bisulfite conversion [18]. Genomic DNA extraction, bisulfite conversion, and Q-MSP analyses were performed using commercially available kits according to the manufacturers’ instructions.

#### Genomic DNA extraction and bisulfite treatment.

FFPE tissue sections were cut into six slices (5 μm thickness), and genomic DNA extraction was performed using the Quick-DNA Miniprep Plus Kit (ZymoResearch, Irvine, CA, USA, Cat no: D4068) according to the manufacturer’s FFPE tissue protocol. Briefly, following deparaffinization, DNA was extracted using *proteinase K* digestion. The concentration and purity of the isolated DNA samples were measured using a NanoDrop 1000 spectrophotometer (Thermo Fisher, Delaware, USA).

Bisulfite modification was performed on 1 µg of DNA isolated from paraffin blocks using the EZ DNA Methylation-Gold^TM^ Kit (Zymo Research, Irvine, CA, USA, Cat no: D5005), according to the manufacturer’s instructions. During bisulfite conversion, unmethylated cytosines are converted to uracil, whereas methylated cytosines remain unchanged. Following this conversion, DNA was subjected to PCR amplification, in which uracil residues are amplified as thymine, while methylated cytosines are retained as cytosine. Since unmethylated cytosine is converted to thymine, all cytosines read in the DNA sequence represent methylation sites. The methylation profiles were determined using forward and reverse methylated primers specifically designed for methylation sites on the *CDO1* promoter. Methylation-specific primers targeting the *CDO1* promoter region were designed to selectively amplify methylated sequences. Primer specificity was verified using in silico analysis, including sequence alignment in FASTA format and validation with relevant NCBI tools, to minimize non-specific amplification.

#### Determination of the amount of DNA required for methylation analysis and preparation of methylation standards.

According to the commercial kit protocol, the optimal amount of DNA per bisulfite treatment ranges from 200 to 500 ng. In this study, 200 ng of DNA was used for each reaction. The required amount of DNA was aliquoted into PCR microplate wells, and the final reaction volume was adjusted to 20 μl, using ultra-pure water. Commercially available fully methylated and unmethylated human DNA standards (Zymo Research, Irvine, CA, USA. Cat. no: D5014) were subjected to bisulfite conversion and used as controls in methylation-specific PCR assays. A series of methylation standards (100, 75, 50, 25, 10, 5, and 0) was prepared by mixing bisulfite-converted methylated and unmethylated DNA in defined proportions. A standard curve was generated using the Ct (cycle threshold) values of these standard and methylation percentages were calculated using Curve Expert software.

#### Quantitative methylation-specific PCR (Q-MSP).

We performed real-time PCR using iQ Supermix (Bio-Rad, Hercules, CA), a real-time PCR System and a real-time PCR Thermal Cycler (Roche Diagnostics) for the detection of *CDO1* promoter methylation levels. Following bisulfite conversion, DNA was amplified by real-time PCR using methylated forward (5’-TTTGGGACGTCGGAGATAAC-3’) and reverse primers (5’-CCAACATTAAAATACCGAAACGTA-3’).

The Q-MSP reaction (20 µl/ well) contained 10 µl of Zymo TaqTM qPCR PreMix (Zymo Research, Irvine, CA, USA. Cat. no: E2055) which includes a hot-start DNA polymerase and buffer system optimized for the amplification of bisulfite-treated DNA, as well as an intercalating double-strand DNA-specific fluorescent dye (SYTO 9®) for sensitive real-time DNA quantification, 2.5 µl of assay-specific forward and reverse primers targeting methylated loci, and bisulfite-treated DNA template together with labelled probes for *CDO1* methylated and unmethylated sequences**.** The PCR reactions were incubated at 95ºC for 10 min, followed by 40 cycles at 95ºC for 15 sec and 55ºC for 1 min. PCRs were performed in duplicate. At the end of the reaction, quantitative analysis of methylated alleles was performed as previously described [19]. The Ct values were converted to percentage methylation using the standard curve.

#### Immunostaining for *CDO1* protein in tissue samples.

FFPE tissue blocks were cut into 5 μm thick sections, and immunohistochemistry was performed as described previously [14]. The sections were incubated with an anti-*CDO1* polyclonal antibody [CUSABIO CSB-PA438445 Cysteine Dioxygenase, Type I [Polyclonal] Conc. 0.1 ml (1: 25–100)]. The secondary antibody reaction was performed using the Histostain-Plus Bulk Kit (Invitrogen, Camarillo, USA). Immunoreactive products were visualized by 3,3-diaminobenzidine (DAB) catalysis.

**Fig 1:**
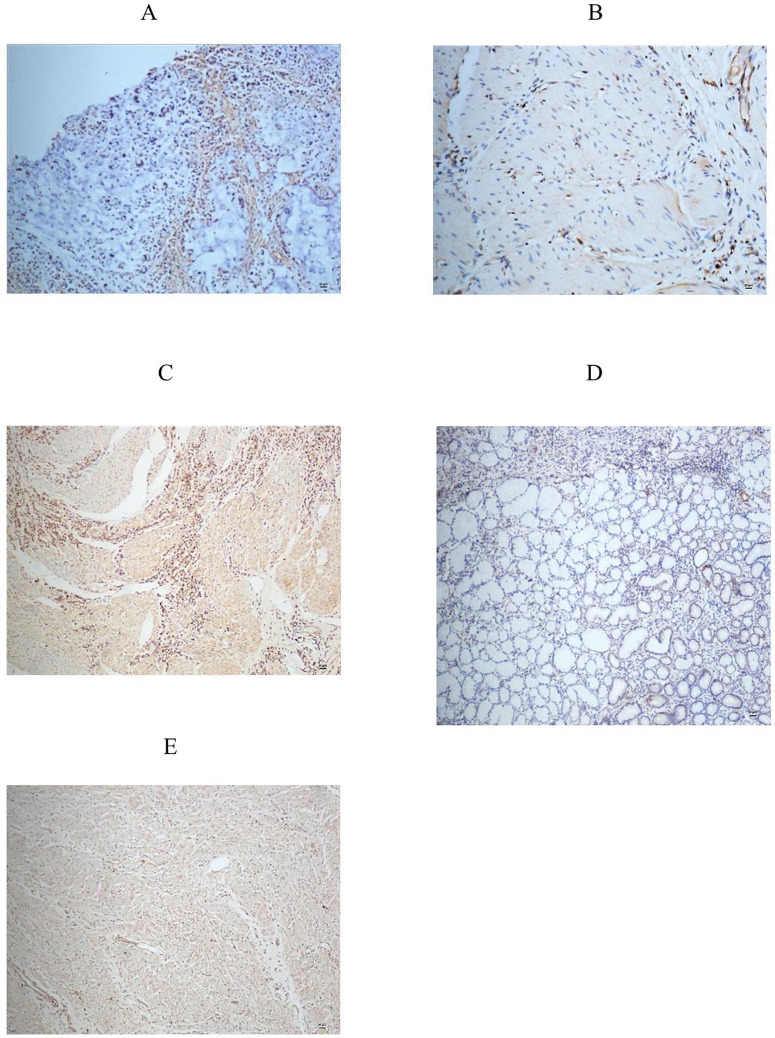
Representative immunostaining of the *CDO1* in the gastric tumors. (A) Low immunoreaction (B) Medium immunoreaction (C) High immunoreaction (D) Negative control staining (E) Positive control staining.

*CDO1* protein levels were evaluated using the immunoreactive-score (IRS) system in primary gastric tumors and biopsy samples of chronic gastritis [20]. Cells were evaluated based on the number of positive cells and the percentage of staining. The *CDO1* immunoreactivity was scored based on the percentages of the stained area. Then, the percentage of the positive cells was scored as follows: 0 (negative, no staining observed), 1 (low [1–40% staining area]), 2 (medium [40–60% staining area]), 3 (high [60–80% staining area]), 4 (very high [80–100% staining area]). Positively stained cells were counted under ×20 magnification in 12 randomly selected fields, and the immunoreactivity scores of each were determined and averaged to obtain a single numerical value. Representative immunostaining of the *CDO1* protein in gastric tumors is presented in Fig 1.

### Statistical analyses

The data were analyzed using SPSS version 22.0 for Windows. The Shapiro-Wilk test was used to assess the normality of data distribution. *CDO1* methylation levels and immunopositivity were not normally distributed; therefore, non-parametric tests, including the Kruskal–Wallis and Mann–Whitney U tests, were used for group comparisons.

For comparisons involving more than two groups, the Kruskal-Wallis was followed by Dunn’s post-hoc test with Bonferroni correction to identify specific group differences. Spearman’s rank correlation analysis was used to evaluated the relationship between variables, as appropriate. The results were presented as median (minimum-maximum).

The optimal cut-off value for % *CDO1* methylation in predicting GC was determined using receiver operating characteristic (ROC) curve analysis, and the “area under the curve” (AUC) was calculated. The optimal cut-off point was defined as the value with the highest combined sensitivity and specificity. A p-value < 0.05 was considered statistically significant.

## Results

The demographic data of the study groups and the characteristics of FFPE samples are presented in Tables 1 and 2, respectively. No significant gender- related difference were observed in *CDO1* gene promoter methylation levels or immunopositivity scores among the the study groups (Table 1). Spearman correlation analysis showed no significant association between age and *CDO1* promoter methylation levels (ρ = −0.079, p = 0.050) or immunopositivity scores (ρ = −0.066, p = 0.520) in the overall cohort.

**Table 1:** The demographic data of the study groups.

	Control (n = 15)	HPN (n = 23)	HPP (n = 17)	GC (n = 45)	p-value
**Sex, n (%)**					
**Female**	6 (40.0)	12 (52.1)	9 (52.9)	17 (37.7)	0.72
**Male**	9 (60.0)	11 (47.8)	8 (47.1)	28 (62.3)	
**Age, median (IQR)**	65 (46.0-77.0)	64 (46.0-77.0)	59 (36.0-84.0)	61 (35.0-86.0)	1.00

Control: Healthy people without *H. pylori* infection, HPN*: H. pylori* negative chronic gastritis, HPP: *H. pylori* positive chronic gastritis, GC: Gastric cancer. IQR: Interquartileranges, p-values correspond to the Kruskal-Wallis test (depending on distribution), and statistically significant differences (p < 0.05) between groups were determined using Dunn’s post-hoc test.

**Table 2:** Characteristics of FFPE samples used for DNA methylation and immunohistochemical analyses.

Tissue	Case no	Cancer	TNM classification	Stage
**Normal**	Control-1	–	–	–
	Control-2	–	–	–
	Control-3	–	–	–
	Control-4	–	–	–
	Control-5	–	–	–
	Control-6	–	–	–
	Control-7	–	–	–
	Control-8	–	–	–
	Control-9	–	–	–
	Control-10	–	–	–
	Control-11	–	–	–
	Control-12	–	–	–
	Control-13	–	–	–
	Control-14	–	–	–
	Control-15	–	–	–
**HPN**	HPN-1	–	–	–
	HPN-2	–	–	–
	HPN-3	–	–	–
	HPN-4	–	–	–
	HPN-5	–	–	–
	HPN-6	–	–	–
	HPN-7	–	–	–
	HPN-8	–	–	–
	HPN-9	–	–	–
	HPN-10	–	–	–
	HPN-11	–	–	–
	HPN-12	–	–	–
	HPN-13	–	–	–
	HPN-14	–	–	–
	HPN-15	–	–	–
	HPN-16	–	–	–
	HPN-17	–	–	–
	HPN-18	–	–	–
	HPN-19	–	–	–
	HPN-20	–	–	–
	HPN-21	–	–	–
	HPN-22	–	–	–
	HPN-23	–	–	–
**HPP**	HPP-1	–	–	–
	HPP-2	–	–	–
	HPP-3	–	–	–
	HPP-4	–	–	–
	HPP-5	–	–	–
	HPP-6	–	–	–
	HPP-7	–	–	–
	HPP-8	–	–	–
	HPP-9	–	–	–
	HPP-10	–	–	–
	HPP-11	–	–	–
	HPP-12	–	–	–
	HPP-13	–	–	–
	HPP-14	–	–	–
	HPP-15	–	–	–
	HPP-16	–	–	–
	HPP-17	–	–	–
**GC**	Cancer-1	+	T4AN2	IIIB
	Cancer-2	+	T4AN0	IIB
	Cancer-3	+	T3N1	IIB
	Cancer-4	+	T1AN0	IA
	Cancer-5	+	T2N2	IIB
	Cancer-6	+	T1BN0	IA
	Cancer-7	+	T4N3B	IIIC
	Cancer-8	+	T4N3	IIIC
	Cancer-9	+	T4AN3A	IIIC
	Cancer-10	+	T3N1	IIB
	Cancer-11	+	T2N0	IB
	Cancer-12	+	T4AN3	IIIC
	Cancer-13	+	T2N2	IIB
	Cancer-14	+	T2N0	IB
	Cancer-15	+	T1N0	IA
	Cancer-16	+	T4AN0	IIB
	Cancer-17	+	T4AN3	IIB
	Cancer-18	+	T4AN3	IIIC
	Cancer-19	+	T1N0	IA
	Cancer-20	+	T4AN3	IIIC
	Cancer-21	+	T4N0	IIB
	Cancer-22	+	T3N3	IIIB
	Cancer-23	+	T3N1	IIB
	Cancer-24	+	T4N3	IIIC
	Cancer-25	+	T3N1	IIB
	Cancer-26	+	T2N2	IIB
	Cancer-27	+	T4N3	IIIC
	Cancer-28	+	T1N0	IA
	Cancer-29	+	T1BN2	IIA
	Cancer-30	+	T4AN0	IIB
	Cancer-31	+	T4AN3	IIIC
	Cancer-32	+	T4AN3	IIIC
	Cancer-33	+	T4AN0	IIB
	Cancer-34	+	T4AN3	IIIC
	Cancer-35	+	T3N0	IIA
	Cancer-36	+	T4AN0	IIB
	Cancer-37	+	T4AN3	IIIC
	Cancer-38	+	T4AN3	IIIC
	Cancer-39	+	T4N3A	IIIC
	Cancer-40	+	T3N0	IIA
	Cancer-41	+	T4AN3	IIIC
	Cancer-42	+	T1AN0	IA
	Cancer-43	+	T1AN0	IA
	Cancer-44	+	T1BN2	IIA
	Cancer-45	+	T4AN3	IIIC

F: Female, M: Male, Control: Healthy people without *H. pylori* infection, HPN: *H. pylori* negative chronic gastritis, HPP: *H. pylori* positive chronic gastritis, GC: Gastric cancer. TNM: Tumor, Node, Metastasis.

The cytoplasmic *CDO1* immunopositivity was observed in all tissue samples. No statistically significant difference were observed among the study groups in terms of *CDO1* immunopositivity scores (Table 3).

**Table 3:** *CDO1* promoter methylation levels and immunopositivity scores of the study groups.

	Control (n = 15)	HPN (n = 23)	HPP (n = 17)	GC (n = 45)	p-value
***CDO1* methylation level**	4.6 (1.7-8.7)	5.05 (1.7-10.6)	10.2 (4.7–22.7) ^**a,b**^	12.6 (1.7–26.4) ^**c,d**^	**0.000**
**Immunopositivity score**	2.33 (1.0-4.0)	2.33 (0.5-4.0)	2.71 (1.67-4.0)	3.0 (1.17-4.0)	0.235

Data of the study groups are given as median (IQR: interquartile ranges). p- values correspond to Kruskal-wallis test (depending on distribution); pairwise comparisons were evaluated using Dunn’s post-hoc test; statistically significant differences are marked with lower cases and bold (p < 0.05). Control: Healthy people without *H. pylori* infection, HPN: *H. pylori* negative chronic gastritis, HPP: *H. pylori* positive chronic gastritis, GC: Gastric cancer, *CDO1: Cysteine dioxygenase-1*.

^a^: p = 0.021 versus the control group.

^b^: p = 0.049 versus the HPN group.

^c^: p = 0.000 versus the control group.

^d^: p = 0.000 versus the HPN group.

**Fig 2:**
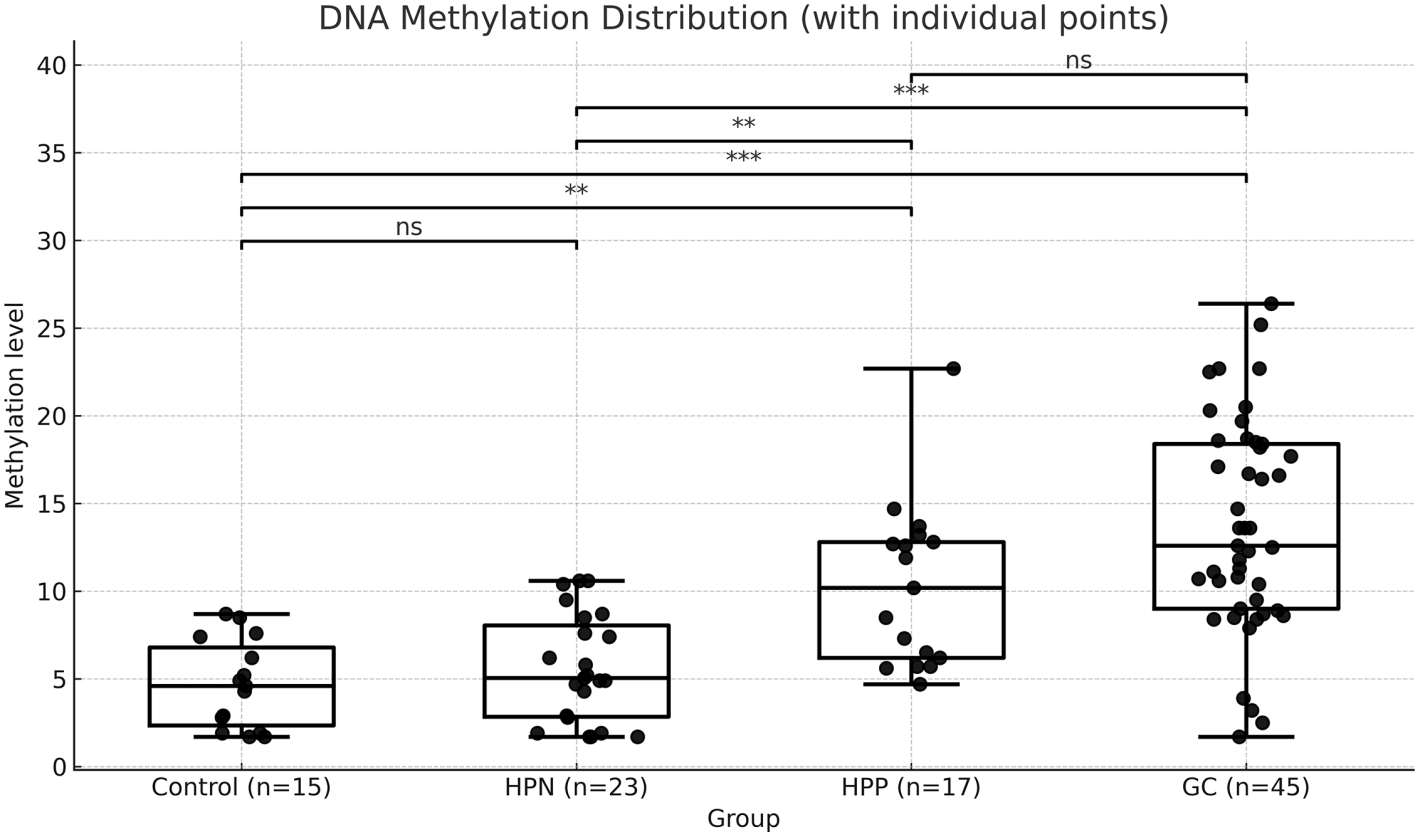
Distribution of the *CDO1* gene promoter methylation levels of the study groups. Control: Healthy people without *H. pylori* infection, HPN: *H. pylori* negative chronic gastritis, HPP: *H. pylori* positive chronic gastritis, GC: Gastric cancer. p-values correspond to Kruskal-Wallis and Dunn post-hoc test (depending on distribution); statistically significant differences are marked with stars. * p < 0.05 ** p < 0.01 *** p < 0.001 ns: not significant.

*CDO1* gene promoter methylation levels were significantly higher in the GC group compared to the HPN chronic gastritis and control groups (p < 0.001) (Table 3). There was no significant difference between the GC group and the HPP chronic gastritis group. The *CDO1* gene promoter methylation levels were significantly higher in the HPP chronic gastritis group than in the HPN chronic gastritis group (p = 0.049) (Table 3). Spearman correlation analysis demonstrated no significant association between *CDO1* promoter methylation levels and immunopositivity scores in the overall cohort (ρ = 0.070, p = 0.495). Similarly, no statistically significant correlations were observed within any of the study groups, including HPN chronic gastritis (ρ = 0.135, p = 0.550), HPP chronic gastritis (ρ = 0.057, p = 0.833), and GC (ρ = −0.249, p = 0.099). The correlation results are summarized in Table 4. The distribution of the *CDO1* gene promoter methylation levels across the study groups is presented in Fig 2.

**Table 4:** Correlation between *CDO1* promoter methylation and immunopositivity scores.

Group	n	Spearman’s ρ	p-value
**Overall**	100	0.070	0.495
**Control**	15	0.121	0.681
**HPN**	23	0.135	0.550
**HPP**	17	0.057	0.833
**GC**	45	−0.249	0.099

**Fig 3:**
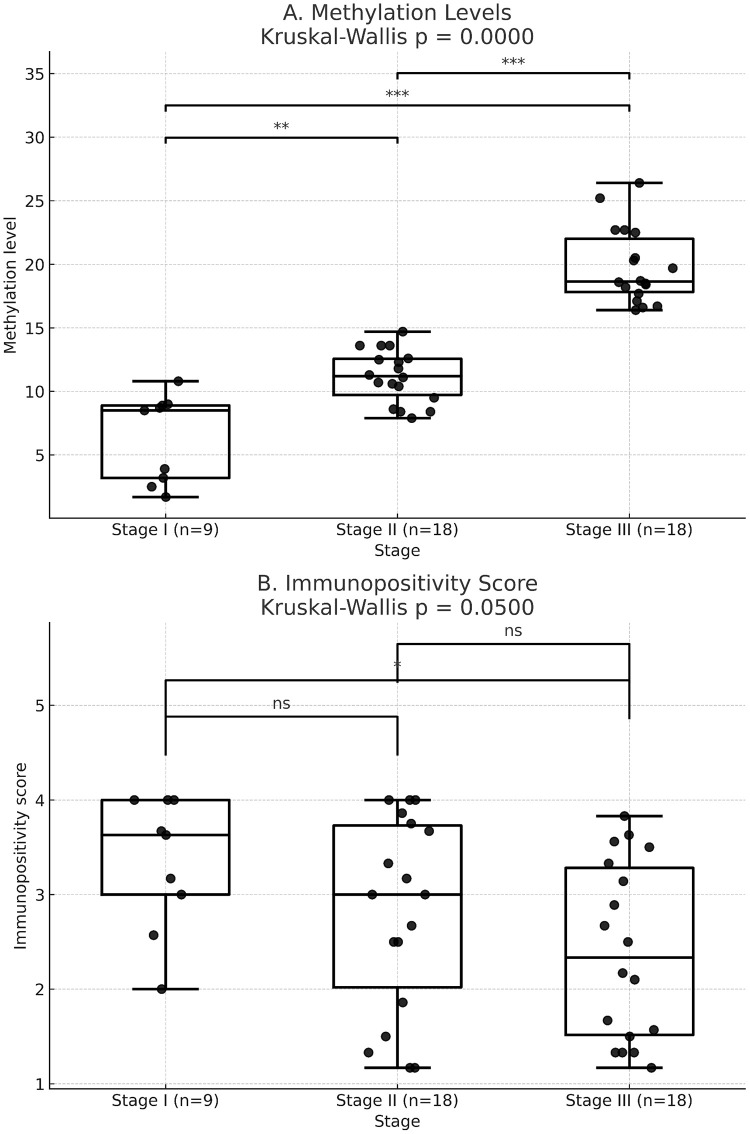
The *CDO1* (A) promoter methylation levels and (B) immunopositivity scores according to the clinical stage of gastric cancer. p-values correspond to Kruskal-Wallis and Dunn post-hoc test (depending on distribution); statistically significant differences are marked with stars. * p < 0.05 ** p < 0.01 *** p < 0.001 ns: not significant.

Spearman’s rank correlation test was used, given the non-parametric nature of the data and the ordinal characteristics of immunohistochemical scoring. No significant correlations were observed between methylation levels and immunohistochemical expression within any subgroup (all p > 0.05). Control: Healthy people without *H. pylori* infection, HPN: *H. pylori* negative chronic gastritis, HPP: *H. pylori* positive chronic gastritis, GC: Gastric cancer,

The GC group was categorized into clinical stages as stage I, stage II, and stage III according to the 2017 AJCC staging system [17]. The methylation levels of the *CDO1* gene promoter increased with advancing clinical stage. There was a significant difference between the stage III and stage I (p < 0.001), as well as between stage III and stage II (p < 0.001) (Fig 3).

**Fig 4:**
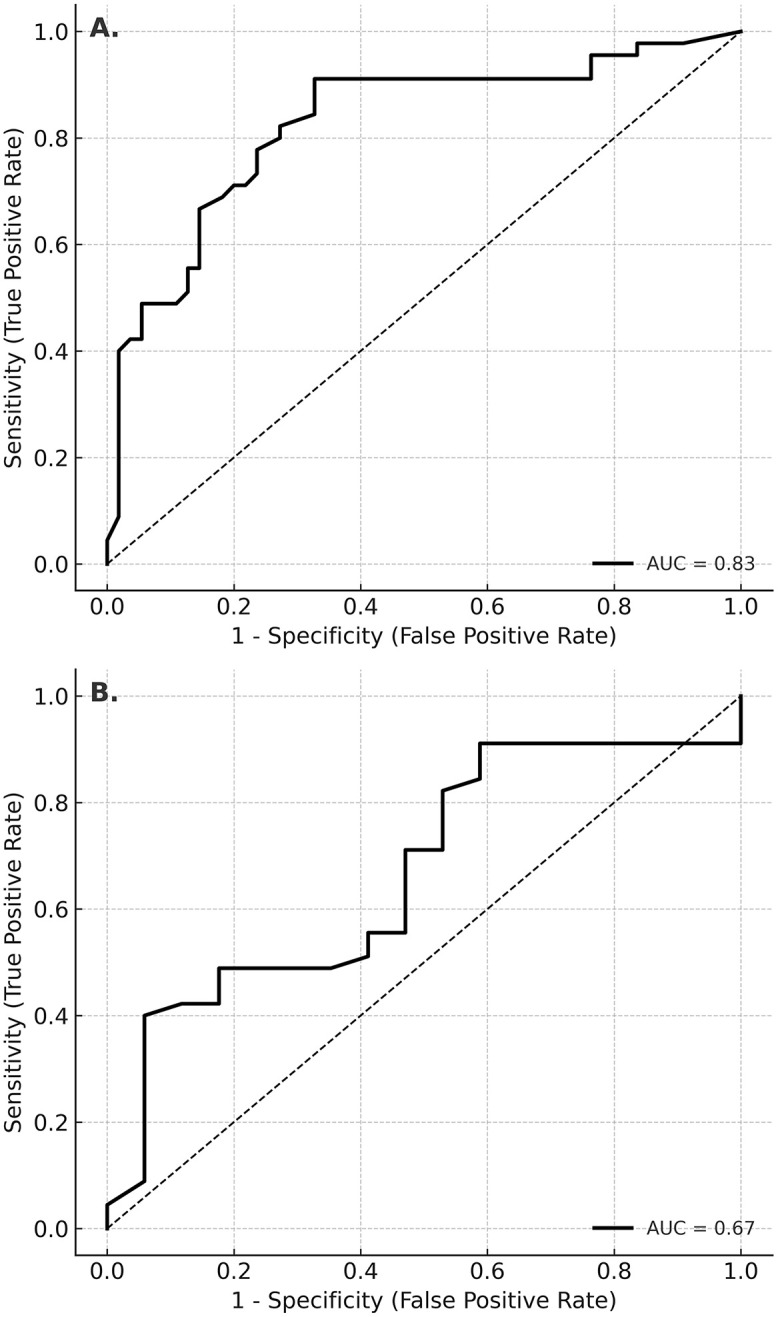
ROC analysis for (A) gastric cancer and non-cancer cases (B) gastric cancer and HPP chronic gastritis cases. (A) AUC: 0.83; Cut-off: 7.90% (91.1% sensitivity and 67.3% specificity). (B) AUC: 0.67; Cut-off: 7.90% (91.1% sensitivity and 41.2% specificity) or cut-off: 16.4% (40.0% sensitivity and 94.1% specificity).

The ROC curve was used to assess the clinical performance of *CDO1* methylation. Two ROC analyses were performed. First, a ROC curve was generated to evaluate the discriminative power of *CDO1* methylation levels between individuals with GC and those without cancer. The model demonstrated good discriminative ability, with an AUC of 0.83 (95% CI: 0.74–0.91, p < 0.0001). This indicates that *CDO1* methylation levels can differentiate between the two groups with acceptable accuracy. The optimal threshold was determined using the Youden Index, which identified a cut-off value of 7.90% DNA methylation (91.1% sensitivity and 67.3% specificity). At this threshold, higher methylation levels were associated with an increased likelihood of GC (Fig 4A). To assess the association between methylation levels and cancer status, a binary logistic regression analysis was conducted (odds ratio: 1.30, 95% CI: 1.16–1.44, p < 0.001). *CDO1* methylation levels were significantly associated with GC status. Specifically, each one-unit increase in methylation level was associated with a 30% increase in the odds of having GC.

Second, a ROC curve was generated to assess the clinical performance of *CDO1* methylation in the differential diagnosis of GC and HPP chronic gastritis (Fig 4B). The ROC analysis demonstrated an AUC of 0.67 (95% CI: 0.512–0.815, p = 0.011), indicating a statistically significant but moderate discriminative ability. Based on the Youden index, the optimal cut-off value was identified as 7.90. At this threshold, the sensitivity and specificity of the test were calculated as 91.1% and 41.2%, respectively. This threshold provides high sensitivity but limited specificity in distinguishing GC from HPP chronic gastritis. As an alternative threshold, the Youden index identified 16.4 as another potential cut-off value. At this threshold, the sensitivity and specificity of the test were calculated as 40.0% and 94.1%, respectively, indicating higher specificity but reduced sensitivity.

## Discussion

In an early study, global DNA methylation of gastric mucosa was evaluated using immunohistochemical detection of 5-methylcytosine. The authors reported that global DNA methylation gradually decreased from normal mucosa to HPP chronic atrophic gastritis, with a significant difference between HPN and HPP subjects. In patients with preneoplastic lesions, global DNA methylation was shown to decrease progressively over time despite *H. pylori* eradication, reaching statistical significance at 10 years compared to baseline [21].

In a more recent study [22], methylation levels of several GC-related genes, including *MOS (*Oncogene MOS, Moloney Murine Sarcoma Virus) miR124a-3, *NKX6−1* (NK6 Homeobox 1), *EMX1 (*Empty Spiracles Homeobox 1), *CDH1*, and *TWIST1* (Twist Family bHLH Transcription Factor 1) were evaluated in non-cancerous gastric mucosa in relation to family history of GC and *H. pylori* infection status. The authors reported that methylation of *MOS* and *CDH1* was associated with gastric carcinogenesis in individuals with a family history of GC, while only *CDH1* methylation decreased following *H. pylori* eradication.

To the best of our knowledge, data on *CDO1* promoter methylation in chronic gastritis with respect to *H. pylori* infection status are limited. In the present study, we demonstrated that *CDO1* promoter methylation levels are significantly higher in HPP chronic gastritis compared to HPN chronic gastritis.

Several studies have reported that specific virulence factors of *H. pylori*, particularly the cytotoxin-associated gene A (*CagA*), can promote aberrant DNA methylation in gastric epithelial cells [23]. *CagA-*positive *H. pylori* strains have been shown to enhance the recruitment of DNA methyltransferases (*DNMTs*) to the promoters of tumor suppressor genes, thereby facilitating epigenetic silencing and potentially accelerating gastric carcinogenesis [24]. Although our study did not stratify patients by *CagA* status, these findings suggest a possible mechanistic link between *H. pylori* infection and *CDO1* promoter hypermethylation, which should be interpreted with caution and warrants further investigation. These findings indicate that *H. pylori* infection may contribute to aberrant methylation of the *CDO1* promoter in gastric mucosa, supporting the involvement of epigenetic mechanisms in inflammation-associated gastric carcinogenesis. Promoter methylation of *CDO1* has attracted increasing attention as a biomarker in GC. Previous studies have demonstrated *CDO1* hypermethylation in peritoneal lavage fluid in advanced-stage GC patients [25], as well as its potential utility in predicting remnant GC following gastrectomy [7], and in identifying patients at risk for metachronous GC [26]. These findings mainly highlight the prognostic value of *CDO1* methylation in advanced disease. In contrast, data regarding its role in early detection and in high-risk conditions such as chronic gastritis, particularly in relation to *H. pylori* infection, remain limited. Therefore, the relationship between *H. pylori* positivity and *CDO1* promoter methylation in chronic gastritis has not yet been clearly established.

In this context, our study provides comparative data on *CDO1* promoter methylation in GC and chronic gastritis with and without *H. pylori* infection. Consistent with previous reports [7,25,26], we observed that *CDO1* promoter methylation levels were significantly increased in gastric tumors and showed an association with clinicopathological features. Moreover, methylation levels were found to increase with advancing tumor stage, suggesting a potential relationship with disease progression. ROC analysis further demonstrated that the *CDO1* methylation has good discriminative ability (AUC = 0.83) and may have potential as a biomarker for GC detection (OR = 1.30, p < 0.001).

A key finding of this study is that while *CDO1* promoter methylation levels were significantly higher in GC compared to HPN chronic gastritis, no significant difference was observed between GC and HPP chronic gastritis. This finding suggests that *H. pylori*-associated chronic inflammation may induce epigenetic alterations that resemble those observed in malignant tissues. In this context, the HPP group appears to exhibit a “GC-like” methylation profile, which may reflect an early epigenetic field effect and a potential pre-neoplastic alteration.

Earlier studies have mainly compared gastric cancer tissues with normal controls, suggesting that CDO1 methylation may have potential diagnostic value. [25–27]. However, such comparisons may not fully reflect the more clinically relevant scenario of distinguishing GC from high-risk conditions such as *H. pylori*-associated chronic gastritis. In this regard, ROC analysis in our study demonstrated that while *CDO1* promoter methylation showed good overall discriminative ability between GC and non-cancer cases (AUC = 0.83), its performance was more limited in the differential diagnosis between GC and HPP chronic gastritis (AUC = 0.67), where the trade-off between sensitivity and specificity became more pronounced.

ROC analysis showed that, at a cut-off value of 7.90, *CDO1* methylation exhibits high sensitivity (91.1%) in detecting cancer cases, but limited specificity (41.2%) in excluding HPP individuals, suggesting that this threshold may be more suitable in contexts where sensitivity is prioritized rather than for definitive discrimination between GC and HPP chronic gastritis, such as a screening tool.

In contrast, at a cut-off value of 16.4, sensitivity decreased (40.0%) while specificity increased (94.1%), reflecting a trade-off between sensitivity and specificity. While this threshold improves specificity, its low sensitivity limits its ability to reliably detect cancer cases. These findings are particularly relevant from a clinical perspective, as HPP chronic gastritis represents a high-risk population in which accurate discrimination from GC is critically important. This threshold may be more appropriate for risk stratification purposes.

Therefore, although higher thresholds may reduce false-positive results, the relatively low sensitivity limits their clinical applicability as standalone confirmatory tools. Taken together, these findings suggest that while *CDO1* methylation may be informative for identifying cancer-related epigenetic alterations in general, its discriminative performance may be insufficient in the more clinically challenging setting of HPP-associated chronic inflammation.

Given that methylation levels increased with advancing tumor stage in our cohort, future studies with larger sample sizes are warranted to determine whether discrimination between HPP chronic gastritis and more advanced GC may be improved.

This highlights a potential limitation of *CDO1* methylation as a standalone biomarker and underscores the need for its evaluation in combination with other clinical or molecular parameters.

A notable finding of the present study is the lack of a significant correlation between *CDO1* promoter methylation levels and protein expression as assessed by immunohistochemistry. The promoter methylation analyses of tumor suppressor genes are generally expected to be associated with transcriptional silencing, and suppressed expression of *CDO1* would therefore be anticipated in the presence of promoter hypermethylation. Promoter hypermethylation of the *CDO1* gene, accompanied by the suppressed expression of *CDO1* protein in immunohistochemistry, was demonstrated in patients with colorectal and gallbladder cancers [14,27].

However, the relationship between *CDO1* promoter methylation and gene expression in GC or chronic gastritis has not been previously investigated. Contrary to expectation, our findings demonstrated no significant correlation between *CDO1* promoter methylation levels and immunopositivity scores. This observation highlights a discrepancy between epigenetic modification and protein expression in this context. Importantly, this lack of correlation does not necessarily indicate the absence of a biological role for *CDO1* methylation, as gene regulation is a complex, multi-layered process. Although promoter hypermethylation is often associated with transcriptional silencing, this relationship is not always linear and may be influenced by additional regulatory mechanisms, including histone modifications, alternative promoter usage, post-transcriptional regulation, and protein stability.

Several factors may contribute to the observed discrepancy. First, different epigenetic mechanisms may act in concert to regulate gene expression. Histone modifications such as acetylation and methylation are closely associated with DNA methylation and may modulate chromatin accessibility, allowing transcription to persist despite promoter methylation [28]. In this context, mRNA expression analysis would be required to clarify whether promoter methylation directly affects transcriptional activity and the absence of such data represents an important limitation of this study. Second post-translational regulation may influence protein levels independently of promoter methylation. The intracellular cysteine concentration plays a critical role in determining the stability and degradation of the *CDO1* protein via the ubiquitin–proteasome system [29]. Therefore, protein levels may not directly reflect promoter methylation status [30]. Third, alternative promoter usage may sustain gene expression even when the canonical promoter region is methylated [31,32]. In addition, tumor heterogeneity and sample composition may also influence the results as clinical tissue samples contain varying proportions of tumor and non-tumor cells, which may differentially contribute to both methylation and protein expression signals.

Importantly, our study was primarily designed to evaluate the potential of *CDO1* promoter methylation as a biomarker, rather than to comprehensively elucidate its functional consequences. In this context, the observed differences in methylation levels between groups remain informative, even in the absence of a direct correlation with protein expression.

Nevertheless, the lack of mRNA expression data, the discrepancy between methylation and protein expression, and potential sample heterogeneity should be considered as key limitations when interpreting the findings. These factors highlight the complexity of epigenetic regulation and should be considered when interpreting biomarker studies based on clinical specimens.

Future studies integrating methylation, mRNA expression, and functional assays are warranted to better elucidate the biological significance of *CDO1* methylation in gastric carcinogenesis.

## Conclusion

The *CDO1* gene promoter levels are increased in GC patients and chronic gastritis patients with *H. pylori* infection. These findings suggest that *CDO1* promoter methylation may reflect early epigenetic alterations associated with gastric carcinogenesis, particularly in HPP chronic gastritis.

Although previous studies have highlighted the potential of *CDO1* methylation as a biomarker for gastric cancer, our findings indicate that its discriminative performance may be more limited in clinically relevant settings, particularly in distinguishing GC from HPP-associated chronic gastritis.

Given its reversible nature, DNA methylation changes in the promoter region of the *CDO1* gene may represent a potential target for early detection strategies. However, this potential should be interpreted with caution and requires further validation.

While this study has several limitations, including the relatively small sample size, the lack of mRNA expression analysis, and the absence of long-term follow-up data, our findings suggest that, although *CDO1* methylation may be informative for identifying cancer-related epigenetic changes, it may be insufficient as a standalone biomarker in high-risk inflammatory conditions.

Further large-scale, prospective studies are warranted to clarify the diagnostic and prognostic value of *CDO1* methylation, particularly in high-risk populations such as patients with *H. pylori*-associated chronic gastritis.

## Supporting information

S1 File Minimal data set. (XLSX)
