# Supplementary material for: A comparative study of CDO1 promoter methylation in gastric cancer and H. pylori-associated chronic gastritis: Implications for diagnostic performance
Source: PLoS One. 2026 Aug 12;21(8):e0335832. doi: 10.1371/journal.pone.0335832 (PMC13465818; doi:10.1371/journal.pone.0335832)
Supplement: Supplementary file 1 [file pone.0335832.s002.docx]

**Journal Requirements:**

1. Please ensure that your manuscript meets PLOS ONE's style requirements, including those for file naming.

**“Our article's compliance with PLOS ONE's style requirements, including file naming, has been reviewed.”**

2. Please state what role the funders took in the study. If the funders had no role, please state: "The funders had no role in study design, data collection and analysis, decision to publish, or preparation of the manuscript." If this statement is not correct, you must amend it as needed. Please include this amended Role of Funder statement in your cover letter; we will change the online submission form on your behalf.

**“Funding statements and their roles are added in the cover letter.”**

3. We note that there is identifying data in the Supporting Information file “S3_File_Data_set.xlsx”. Due to the inclusion of these potentially identifying data, we have removed this file from your file inventory. Prior to sharing human research participant data, authors should consult with an ethics committee to ensure data are shared in accordance with participant consent and all applicable local laws. Please remove or anonymize all personal information (<specific identifying information in file to be removed>), ensure that the data shared are in accordance with participant consent, and re-upload a fully anonymized data set. Please note that spreadsheet columns with personal information must be removed and not hidden as all hidden columns will appear in the published file.

**“S3_File_Data_set.xlsx corrected. Ages and genders of the participants are removed.”**

4. If the reviewer comments include a recommendation to cite specific previously published works, please review and evaluate these publications to determine whether they are relevant and should be cited. There is no requirement to cite these works unless the editor has indicated otherwise.

“**The reviewers did not recommend citing any specific previously published works.”**

**Additional Editor Comments:**

When responding to Reviewers' comments, the authors should also generally improve the methodology, including specifying the criteria used to diagnose samples used in the study, and also the sampling procedure. The authors should also highlight all the limitations to this study, such as when suggesting their novel test as confirmatory with only 40% sensitivity.

**Author's Response:**

We thank the Editor for these valuable and constructive comments. In response, we have carefully revised the manuscript to improve methodological clarity and to more explicitly address the study limitations.

**First,** the **Methods section** has been revised to provide clearer details regarding the diagnostic criteria and sampling procedures. Specifically, we have clarified that ***H. pylori* infection status was determined by histopathological evaluation of gastric biopsy specimens using modified Giemsa staining,** and **we have further elaborated on the patient selection and sample processing procedures.**

**Second,** we have expanded **the Discussion section** to more explicitly acknowledge the **limitations of the study.** In particular, we now clearly state the **lack of mRNA expression data, the relatively small sample size, the absence of long-term follow-up, and the potential impact of sample heterogeneity.**

**Finally,** we have revised the interpretation of the **ROC analysis** to avoid overstatement of clinical applicability. The discussion of the alternative cut-off value with lower sensitivity has been substantially tempered, and it is now clearly stated that ***CDO1* methylation alone may not be sufficient as a standalone diagnostic or confirmatory tool and should be interpreted with caution.**

We believe that these revisions have significantly improved the clarity, transparency, and clinical interpretation of the study.

**Response to Reviewer_1**

We believe that these revisions have strengthened the manuscript and improved the clarity and balance of our interpretations. We thank the reviewer again for these valuable suggestions, which have significantly contributed to improving the quality of our work.

Section for INTRODUCTION

**Reviewer comment:**

This section contains some abbreviations that are not fully explained when first mentioned in the text, such as CDO1. The authors should use the full forms first, then use the abbreviations. In addition, the authors clearly explain the study's objective.

**Author's Response:**

We would like to thank the reviewer for this careful reading and the positive feedback regarding the clarity of our study's objective.

We have addressed the concern regarding abbreviations throughout the Introduction. **Specifically, the full name for CDO1 (Cysteine Dioxygenase Type 1) and other relevant terms have been provided upon their first mention.** We have also performed a thorough check of the entire manuscript to ensure all abbreviations are consistently defined.

Changes in the Manuscript:

The full forms of abbreviations have been added in the Introduction section and the entire article.

Section for METHODS

**Reviewer comment:**

The strength of the study is that written informed consent was obtained from the patients, despite the study being retrospective. The authors should explain why they chose to evaluate the pathological samples, including at least 30% of tumor cells. Why this ratio? According to which guideline? Apart from these, the methods section is comprehensive and thoroughly explains each step used in the research.

**Author's Response:**

We thank the reviewer for this insightful and constructive comment. We agree that clarification of the tumor cellularity threshold is important for methodological transparency.

Tumor cellularity is a critical factor in molecular pathology, as the presence of stromal, inflammatory, or normal epithelial cells may dilute tumor-derived DNA and reduce the sensitivity of molecular assays. Therefore, assessment of tumor cell content by a pathologist prior to molecular testing is widely recommended to ensure that the analyzed nucleic acids adequately represent the tumor component [1,2].

International molecular pathology guidelines, including those from the College of American Pathologists (CAP), the International Association for the Study of Lung Cancer (IASLC), and the Association for Molecular Pathology (AMP), emphasize the importance of evaluating tumor cellularity before molecular analysis to avoid false-negative results due to insufficient tumor DNA. <https://www.cap.org/protocols-and-guidelines/cap-guidelines/current-cap-guidelines/molecular-testing-guideline-for-the-selection-of-lung-cancer-patients-for-treatment-with-tyrosine-kinase-inhibitors?utm_source=chatgpt.co>. [1]. However, these guidelines do not define a strict universal cut-off, but rather recommend ensuring sufficient tumor content for reliable detection.

In clinical molecular pathology practice, tumor cellularity thresholds in the range of approximately 20–30% are commonly adopted for PCR-based assays, including methylation analyses, to ensure reliable detection of tumor-specific molecular alterations although no universally accepted cut-off has been formally established [1–5]. Although PCR-based methods can theoretically detect low levels of altered DNA under controlled conditions, the reliability of detection in formalin-fixed paraffin-embedded (FFPE) clinical samples is strongly influenced by tumor purity, sample heterogeneity, and pre-analytical variables [3–5]. When tumor cellularity is low, the increased proportion of non-tumoral DNA may dilute tumor-specific signals and compromise analytical sensitivity.

Based on these widely accepted principles and previous literature, we included only tissue sections containing at least 30% tumor cells, as evaluated by an experienced pathologist. This threshold was selected as a conservative and commonly used criterion to minimize contamination by non-neoplastic cells and to ensure adequate tumor-derived DNA for the quantitative methylation-specific PCR analysis performed in this study.

**References**

1. Lindeman NI, Cagle PT, Aisner DL, Arcila ME, Beasley MB, Bernicker EH, Colasacco C, Dacic S, Hirsch FR, Kerr K, Kwiatkowski DJ, Ladanyi M, Nowak JA, Sholl L, Temple-Smolkin R, Solomon B, Souter LH, Thunnissen E, Tsao MS, Ventura CB, Wynes MW, Yatabe Y. Updated Molecular Testing Guideline for the Selection of Lung Cancer Patients for Treatment With Targeted Tyrosine Kinase Inhibitors: Guideline From the College of American Pathologists, the International Association for the Study of Lung Cancer, and the Association for Molecular Pathology. Arch Pathol Lab Med. 2018 Mar;142(3):321-346. doi: 10.5858/arpa.2017-0388-CP. Epub 2018 Jan 22. PMID: 29355391.
2. van Krieken JH, Normanno N, Blackhall F, Boone E, Botti G, Carneiro F, Celik I, Ciardiello F, Cree IA, Deans ZC, Edsjö A, Groenen PJ, Kamarainen O, Kreipe HH, Ligtenberg MJ, Marchetti A, Murray S, Opdam FJ, Patterson SD, Patton S, Pinto C, Rouleau E, Schuuring E, Sterck S, Taron M, Tejpar S, Timens W, Thunnissen E, van de Ven PM, Siebers AG, Dequeker E. Guideline on the requirements of external quality assessment programs in molecular pathology. Virchows Arch. 2013 Jan;462(1):27-37. doi: 10.1007/s00428-012-1354-4. Epub 2012 Dec 19. PMID: 2325035
3. Jennings LJ, Arcila ME, Corless C, Kamel-Reid S, Lubin IM, Pfeifer J, Temple-Smolkin RL, Voelkerding KV, Nikiforova MN. Guidelines for Validation of Next-Generation Sequencing-Based Oncology Panels: A Joint Consensus Recommendation of the Association for Molecular Pathology and College of American Pathologists. J Mol Diagn. 2017 May;19(3):341-365. doi: 10.1016/j.jmoldx.2017.01.011. Epub 2017 Mar 21. PMID: 28341590; PMCID: PMC6941185.
4. Schmitz, M., Wunsch, K., Hoyer, H. et al. Performance of a methylation specific real-time PCR assay as a triage test for HPV-positive women. Clin Epigenet 9, 118 (2017). <https://doi.org/10.1186/s13148-017-0419-2>
5. Roy-Chowdhuri S, Stewart J. Preanalytic Variables in Cytology: Lessons Learned From Next-Generation Sequencing-The MD Anderson Experience. Arch Pathol Lab Med. 2016 Nov;140(11):1191-1199. doi: 10.5858/arpa.2016-0117-RA. Epub 2016 Jun 22. PMID: 27333361.

To address the reviewer’s comment, we have now clarified this point in the **METHODS** section of the revised manuscript (Materials and Methods: Study design and clinical samples: Last paragraph ) as follows:

**“Only tissue sections containing at least 30% tumor cells, as assessed by an experienced pathologist, were included in the analysis to ensure sufficient tumor DNA for reliable PCR-based methylation analysis, in line with commonly adopted practices in molecular pathology [1,5].”**

Section for RESULTS

**Reviewer comment:**

Nomenclature confusion: The authors should use a single nomenclature for the group without gastritis and H pylori. However, they label them “normal” in one table and “control” in another.

All tables should include an explanation of the abbreviations as a footnote at the bottom. For example, the CDO1 explanation is missing in Table 3.

Apart from these, the results section includes sufficient illustrative examples and robust statistical analysis methods for this research.

In Table 1, the spelling of Famale should be corrected to Female.

**Author's Response:**

In response to the nomenclature inconsistency, we have revised all tables and the main text to ensure the use of a single, consistent term. The group without gastritis and H. pylori infection is now uniformly referred to as the **“control”** group throughout the manuscript.

We also agree that clear definition of abbreviations improves readability. Accordingly, we have added explanatory footnotes for all abbreviations in each table. Specifically, the abbreviation **“*CDO1*” has now been defined in Table 3,** and similar clarifications have been provided wherever needed.

In addition, the typographical error in Table 1 has been corrected, and “Famale” has been revised to **“Female”.**

These changes have been incorporated into the revised manuscript (Tables 1–3) to improve clarity, consistency, and overall presentation.

**Section for DISCUSSION**

**Reviewer comment:**

Although the central finding indicates that CDO1 gene promoter methylation levels are significantly higher in the GC and HPP groups, there is no significant difference in CDO1 positivity between the groups. This disconnect fundamentally challenges the functional impact of the observed hypermethylation. While the authors discuss the potential reasons for this in the discussion section, the lack of mRNA expression data remains a significant limitation. Without demonstrating that promoter methylation actually leads to transcriptional silencing of CDO1, the study's core argument is notably weakened.

The second major concern is that the ROC analysis for differentiating GC from HPP-chronic gastritis, the authors suggest an alternative cut-off value of 164%, which yields a sensitivity of 40% and a specificity of 94%. Proposing a biomarker test with only a 40% sensitivity for cancer detection is clinically problematic since it would result in a high rate of false negatives. The rationale for suggesting this threshold as a “confirmatory tool” needs stronger clinical justification, or the limitations of this test’s diagnostic value should be stated more explicitly.

**Author's Response:**

We sincerely thank the reviewer for this insightful and important comment.

**First;** **we fully agree that demonstrating the functional consequences of promoter methylation at the mRNA level would strengthen the biological interpretation of our findings.** In the present study, we observed significantly increased CDO1 promoter methylation levels in both gastric cancer (GC) and *H. pylori*-positive chronic gastritis groups; however, this was not paralleled by a significant difference in protein expression as assessed by immunohistochemistry. **As correctly pointed out, this discrepancy limits the ability to directly infer transcriptional silencing based solely on methylation data.**

**Importantly, our study was primarily designed to evaluate the potential of CDO1 promoter methylation as a biomarker rather than to comprehensively investigate its functional consequences.** In this context, the observed differences in methylation levels between groups remain informative. **Nevertheless, we have now more explicitly acknowledged that the lack of mRNA expression data represents an important limitation of the study.**

We also acknowledge that the relationship between promoter methylation and gene expression is complex and not always linear. Gene expression may be influenced by multiple regulatory mechanisms, including histone modifications, alternative promoter usage, post-transcriptional regulation, and protein stability (Bu S, et al; Rauluseviciute I, et al). Therefore, the absence of a direct correlation between methylation status and protein expression does not necessarily exclude a biological role for CDO1 methylation.

In the revised manuscript, we have strengthened the Discussion section by (i) clearly stating the absence of mRNA expression data as a limitation, (ii) further tempering the interpretation of the functional implications of methylation, and (iii) emphasizing the need for future studies integrating methylation, transcriptional, and functional analyses to clarify the biological significance of CDO1 promoter methylation (Discussion: 13 -18. paragraph ).

**Second**; We agree that a sensitivity of **40% is relatively low for cancer detection and may result in a substantial number of false-negative cases if used as a standalone diagnostic tool.**

In the original version, the interpretation of this threshold may have been overly suggestive of clinical applicability. In response to the reviewer’s comment, **we have substantially revised and tempered this interpretation in the Discussion section.**

Specifically, we now emphasize that although different cut-off values illustrate the trade-off between sensitivity and specificity, the higher threshold (16.4) is associated with limited sensitivity and may result in a considerable number of false-negative cases.

**Importantly, we have removed the previous implication that this threshold could be used as a “confirmatory tool” and instead clearly state that CDO1 methylation alone is insufficient as a standalone diagnostic approach, particularly in the clinically relevant distinction between GC and HPP-chronic gastritis.**

**Furthermore, we now highlight that while CDO1 methylation demonstrates good overall discriminative ability between GC and non-cancer cases, its performance is more limited in distinguishing GC from HPP-associated chronic gastritis,** which represents a more clinically challenging and relevant scenario (Discussion: 10 -12. paragraph ).

We appreciate the reviewer’s comment, which has significantly improved the clarity, balance, and clinical interpretation of our findings.

**Final reviewer comment**
As a clinician in internal medicine, I would be pleased to have a new emerging option to detect gastric cancers at earlier stages, thereby prolonging patients' survival. This study clinically supports this idea; however, there are major limitations. There are substantial discrepancies between the molecular findings and protein expression that remain unresolved. Additionally, concerns regarding sample purity and the proposed clinical utility of the statistical cut-offs need to be addressed before the manuscript can be considered for publication. Therefore, I recommend a major revision for this article.

**Author's Response:**

We sincerely thank the reviewer for this comprehensive and constructive evaluation of our study. **We appreciate the reviewer’s recognition of the potential clinical relevance of our findings, particularly in the context of early detection of gastric cancer.**

**We fully acknowledge the key limitations highlighted by the reviewer and have carefully revised the manuscript to address each of these concerns in a more explicit and balanced manner.** Specifically, the discrepancy between promoter methylation and protein expression, the potential impact of sample purity, and the clinical interpretation of the proposed cut-off values have now been more clearly and systematically addressed.

**In addition, we have refined the overall interpretation of our findings in light of the existing literature. While previous studies have highlighted CDO1 methylation as a promising biomarker for gastric cancer, these have largely been based on comparisons between cancer and normal tissues (**Ushiku H, et al; Kubota Y, et al; Kojima K, et al)**. In contrast, our study evaluates a more clinically relevant scenario, demonstrating that the discriminative performance of CDO1 methylation is more limited in distinguishing GC from H. pylori-associated chronic gastritis. This perspective has been incorporated into the “revised Discussion” to better contextualize our findings.**

In addition, the title has been revised as **“A comparative study of CDO1 promoter methylation in gastric cancer and H. pylori-associated chronic gastritis: Implications for diagnostic performance”** to better reflect the scope of the study and the context-dependent interpretation of CDO1 methylation.

Accordingly:

• **The discrepancy between CDO1 promoter methylation and protein expression has been more thoroughly discussed, and we now explicitly emphasize that the lack of mRNA expression data represents an important limitation in interpreting the functional impact of methylation** (Discussion: 13 -18. paragraph ).

• **Tumor cellularity and sample composition have been clarified in the Methods section, including justification of the ≥30% tumor cell threshold in accordance with commonly adopted practices in molecular pathology** (Materials and Methods: Study design and clinical samples: Last paragraph )

• **The clinical interpretation of ROC-derived cut-off values has been substantially revised. In particular, we have tempered our previous statements and now clearly state that the higher cut-off value, despite improved specificity, is associated with limited sensitivity and may lead to false-negative results. Therefore, CDO1 methylation is no longer presented as a standalone diagnostic or confirmatory tool, but rather as a marker whose performance is context-dependent and more limited in the clinically relevant distinction between GC and H. pylori-associated chronic gastritis** (Discussion: 5 -12. paragraph ).

**Taken together, these revisions have strengthened the transparency, balance, and clinical interpretation of our study, and we believe they address the reviewer’s concerns.**

**Referances**

- Bu S, Ye T, Gao H, Song H, Zhu Y. Histone methylation and acetylation in cancer: mechanism, progression, and targets. Oncologie. 2024;27(1):29-43. doi: <https://doi.org/10.1515/oncologie-2024-0324>.
- Rauluseviciute I, Drabløs F, Rye MB. DNA hypermethylation associated with upregulated gene expression in prostate cancer demonstrates the diversity of epigenetic regulation. BMC Med Genomics. 2020;13(1):6. Epub 20200108. doi: 10.1186/s12920-020-0657-6. PubMed PMID: 31914996; PubMed Central PMCID: PMCPMC6950795.
- Ushiku H, Yamashita K, Ema A, Minatani N, Kikuchi M, Kojo K, et al. DNA diagnosis of peritoneal fluid cytology test by CDO1 promoter DNA hypermethylation in gastric cancer. Gastric Cancer. 2017;20(5):784-92. Epub 20170227. doi: 10.1007/s10120-017-0697-6. PubMed PMID: 28243814.
- Kubota Y, Tanabe S, Azuma M, Horio K, Fujiyama Y, Soeno T, et al. Predictive Significance of Promoter DNA Methylation of Cysteine Dioxygenase Type 1 (CDO1) in Metachronous Gastric Cancer. J Gastric Cancer. 2021;21(4):379-91. Epub 20211207. doi: 10.5230/jgc.2021.21.e35. PubMed PMID: 35079440; PubMed Central PMCID: PMCPMC8753284.
- Kojima K, Nakamura T, Ohbu M, Katoh H, Ooizumi Y, Igarashi K, et al. Cysteine dioxygenase type 1 (CDO1) gene promoter methylation during the adenoma-carcinoma sequence in colorectal cancer. PLoS One. 2018;13(5):e0194785. Epub 20180510. doi: 10.1371/journal.pone.0194785. PubMed PMID: 29746493; PubMed Central PMCID: PMCPMC5944981.

**Response to Reviewer_2**

**Reviewer comments**

1. In the introduction section, epidemiology of gastric cancer could’ve been added to highlight the disease’s significance.

**Author's Response**

We thank the reviewer for this valuable suggestion. We agree that including epidemiological data would strengthen the context and highlight the clinical significance of gastric cancer.

Accordingly, we have revised the Introduction section as follows: To include a brief overview of the global incidence and mortality of gastric cancer, emphasizing its continued burden and clinical importance. (Introduction: First paragraph)

*“Gastric cancer is the fifth most common malignancy worldwide and remains a leading cause of cancer-related mortality, according to Global Cancer Statistics (GLOBOCAN) 2022 [Bray et al., 2024]. The presence of precancerous conditions, particularly Helicobacter pylori infection, is a well-established risk factor for gastric carcinogenesis. Although endoscopic examination plays a crucial role in the early detection of gastric cancer, its invasive nature and limited patient compliance restrict its widespread use in screening and follow-up. Therefore, there is a growing need for non-invasive approaches that can be utilized for early diagnosis, risk stratification, and disease monitoring, as well as for identifying potential molecular biomarkers and therapeutic targets.”*

Ref: Bray F, Laversanne M, Sung H, Ferlay J, Siegel RL, Soerjomataram I, et al. Global cancer statistics 2022: GLOBOCAN estimates of incidence and mortality worldwide for 36 cancers in 185 countries. CA Cancer J Clin. 2024;74(3):229-263.

**Reviewer comments**

2. The methodology section of this study lacks specific information regarding the diagnostic criteria used to confirm *H.Pylori* status of the patients included in the study cohort.

**Author's Response**

We thank the reviewer for pointing this out. We have clarified the methodology regarding H. pylori determination in the revised manuscript. Specifically, H. pylori infection status was assessed on biopsy specimens using histopathological evaluation with modified Giemsa staining, which is widely considered the gold standard for detecting H. pylori in clinical practice. This ensures accurate classification of patients into H. pylori-positive and H. pylori-negative chronic gastritis groups. **The revised sentence has been integrated into the Methods section as follows:** (Material Method: Study design and clinical samples: 3. Paragraph)

*“H. pylori infection status in chronic gastritis patients was determined by histopathological examination of gastric biopsy specimens using modified Giemsa staining, a widely accepted routine diagnostic method in clinical pathology practice.”*

This addition provides transparency and methodological rigour regarding the determination of *H. pylori* status in our study cohort.

**Reviewer comments**

3. The authors reported a significant association between H. Pylori infection and CDO1 promoter hypermethylation. However, the manuscript lacks any discussion of bacterial virulence factors, particularly CagA that have been reported to accelerate the recruitment of DNA methyltransferases (DNMTs) to tumor suppressor promoters.

**Author's Response**

We thank the reviewer for this important suggestion. We agree that H. pylori virulence factors, particularly CagA, may play a mechanistic role in promoting aberrant DNA methylation. To address this, we have added the following discussion in the manuscript (Discussion: 4. Paragraph)

*“Several studies have reported that specific virulence factors of H. pylori, particularly the cytotoxin-associated gene A (CagA), can promote aberrant DNA methylation in gastric epithelial cells [Zhang et al., 2016]. CagA-positive H. pylori strains have been shown to enhance the recruitment of DNA methyltransferases (DNMTs) to the promoters of tumor suppressor genes, thereby facilitating epigenetic silencing and potentially accelerating gastric carcinogenesis [Luan et al., 2025]. Although our study did not stratify patients by CagA status, these findings suggest a possible mechanistic link between H. pylori infection and CDO1 promoter hypermethylation, which warrants further investigation.”*

This addition contextualizes our findings within the known literature on *H. pylori* virulence factors while clarifying that CagA status was not assessed in our cohort, and highlights a potential direction for future studies.

- Zhang BG, Hu L, Zang MD, Wang HX, Zhao W, Li JF, Su LP, Shao Z, Zhao X, Zhu ZG, Yan M, Liu B. Helicobacter pylori CagA induces tumor suppressor gene hypermethylation by upregulating DNMT1 via AKT-NFκB pathway in gastric cancer development. Oncotarget. 2016 Mar 1;7(9):9788-800. doi: 10.18632/oncotarget.7125. PMID: 26848521; PMCID: PMC4891084.
- Luan, M., Zhu, W., Feng, Z. *et al.* *Helicobacter pylori*-induced aberrant methylation of ID4 mediated by DNMT3B drives gastric cancer progression via DEC1-SHH signaling pathway. *Cell Death Dis* **16**, 713 (2025). https://doi.org/10.1038/s41419-025-08042-9

**Reviewer comments**

4. In the results section, Table 3 corresponds to the comparison of methylation levels and IHC scores across various groups. The authors performed Kruskal Wallis test, however they also reported p values for individual group comparisons. But Kruskal Wallis test does not provide pairwise comparison, so it needs to be specified that which test was used to get these significance levels.

**Author's Response**

We thank the reviewer for this important comment. We agree that the Kruskal-Wallis test only indicates an overall difference among groups and does not identify which specific groups differ. Accordingly, **we have clarified in the Statistical Analysis section that** the Kruskal-Wallis test was used for overall group comparisons and that, **when the overall result was significant, Dunn’s post-hoc test with Bonferroni correction** was applied for pairwise comparisons. We have also revised the footnotes of **Table 3** accordingly and updated **Table 1** for consistency in the presentation of the statistical methods throughout the manuscript.

**Reviewer comments**

5. The authors reported that there was no significant correlation between CDO1 gene promoter methylation levels and CDO1 immunopositivity in the study groups. However, the statistical analysis to support this is missing (i.e; Spearman correlation or Pearson’s correlation).

**Author's Response**

We thank the reviewer for this valuable comment. We would like to clarify that correlation analyses between CDO1 promoter methylation levels and immunohistochemical expression scores had already been performed during the initial analysis. However, as no statistically significant associations were identified, these results were not included in the original manuscript.

**In response to the reviewer’s suggestion, we have now incorporated these analyses into the revised manuscript for completeness and transparency.** Spearman’s rank correlation test was used, given the non-parametric nature of the data and the ordinal characteristics of immunohistochemical scoring. (Results: 1. And 3. Paragraph)

*The results confirmed the absence of a significant correlation in the overall cohort (ρ = 0.070, p = 0.495), as well as within the subgroups, including H. pylori-negative chronic gastritis (ρ = 0.135, p = 0.550), H. pylori-positive chronic gastritis (ρ = 0.057, p = 0.833), and gastric cancer (ρ = −0.249, p = 0.099).*

**These results have now been added to the Results section and are also presented in a newly created table (Table 4) to improve clarity.**

**Additionally,** Spearman correlation analysis age and *CDO1* promoter methylation levels (ρ = -0.079, p = 0.040) with immunopositivity scores (ρ = -0.066, p = 0.520) in the overall cohort was added and in the results section was revised.

**Reviewer comments**

6. The manuscript should be thoroughly checked and corrected for grammatical mistakes.

**Author's Response**

Finally; we have carefully reviewed the entire manuscript and corrected grammatical and typographical errors to improve clarity and readability.
