# Supplementary material for: A comparative study of CDO1 promoter methylation in gastric cancer and H. pylori-associated chronic gastritis: Implications for diagnostic performance
Source: PLoS One. 2026 Aug 12;21(8):e0335832. doi: 10.1371/journal.pone.0335832 (PMC13465818; doi:10.1371/journal.pone.0335832)
Supplement: Peer review history

# Peer review: Decision Letter 0

**DOI:** 10.1371/journal.pone.0335832.r001

**Authors:** Elingarami Sauli

10 Mar 2026

Dear Dr.  Kankaya,

Thank you for submitting your manuscript to PLOS ONE. After careful consideration, we feel that it has merit but does not fully meet PLOS ONE’s publication criteria as it currently stands. Therefore, we invite you to submit a revised version of the manuscript that addresses the points raised during the review process.

Please submit your revised manuscript by Apr 24 2026 11:59PM. If you will need more time than this to complete your revisions, please reply to this message or contact the journal office at plosone@plos.org. When you're ready to submit your revision, log on to https://www.editorialmanager.com/pone/ and select the 'Submissions Needing Revision' folder to locate your manuscript file.

- A letter that responds to each point raised by the academic editor and reviewer(s). You should upload this letter as a separate file labeled 'Response to Reviewers'.
- A marked-up copy of your manuscript that highlights changes made to the original version. You should upload this as a separate file labeled 'Revised Manuscript with Track Changes'.
- An unmarked version of your revised paper without tracked changes. You should upload this as a separate file labeled 'Manuscript'.

If you would like to make changes to your financial disclosure, please include your updated statement in your cover letter. Guidelines for resubmitting your figure files are available below the reviewer comments at the end of this letter.

If applicable, we recommend that you deposit your laboratory protocols in protocols.io to enhance the reproducibility of your results. Protocols.io assigns your protocol its own identifier (DOI) so that it can be cited independently in the future. For instructions see: https://journals.plos.org/plosone/s/submission-guidelines#loc-laboratory-protocols. Additionally, PLOS ONE offers an option for publishing peer-reviewed Lab Protocol articles, which describe protocols hosted on protocols.io. Read more information on sharing protocols at https://plos.org/protocols?utm_medium=editorial-email&utm_source=authorletters&utm_campaign=protocols.

We look forward to receiving your revised manuscript.

Kind regards,

Elingarami Sauli, PhD

Academic Editor

PLOS One

Journal Requirements:

When submitting your revision, we need you to address these additional requirements.

1.Please ensure that your manuscript meets PLOS ONE's style requirements, including those for file naming. The PLOS ONE style templates can be found at

https://journals.plos.org/plosone/s/file?id=wjVg/PLOSOne_formatting_sample_main_body.pdf and

https://journals.plos.org/plosone/s/file?id=ba62/PLOSOne_formatting_sample_title_authors_affiliations.pdf

2. Thank you for stating the following financial disclosure:

“This work was supported by Istanbul University - Cerrahpaşa Scientific Research Projects Coordination Unit. Project number: 35156.”

Please state what role the funders took in the study.  If the funders had no role, please state: "The funders had no role in study design, data collection and analysis, decision to publish, or preparation of the manuscript."

If this statement is not correct you must amend it as needed.

Please include this amended Role of Funder statement in your cover letter; we will change the online submission form on your behalf.

3. We note that there is identifying data in the Supporting Information file “S3_File_Data_set.xlsx”. Due to the inclusion of these potentially identifying data, we have removed this file from your file inventory. Prior to sharing human research participant data, authors should consult with an ethics committee to ensure data are shared in accordance with participant consent and all applicable local laws.

Data sharing should never compromise participant privacy. It is therefore not appropriate to publicly share personally identifiable data on human research participants. The following are examples of data that should not be shared:

-Name, initials, physical address

-Ages more specific than whole numbers

-Internet protocol (IP) address

-Specific dates (birth dates, death dates, examination dates, etc.)

-Contact information such as phone number or email address

-Location data

-ID numbers that seem specific (long numbers, include initials, titled “Hospital ID”) rather than random (small numbers in numerical order)

Data that are not directly identifying may also be inappropriate to share, as in combination they can become identifying. For example, data collected from a small group of participants, vulnerable populations, or private groups should not be shared if they involve indirect identifiers (such as sex, ethnicity, location, etc.) that may risk the identification of study participants.

Additional guidance on preparing raw data for publication can be found in our Data Policy (https://journals.plos.org/plosone/s/data-availability#loc-human-research-participant-data-and-other-sensitive-data) and in the following article: http://www.bmj.com/content/340/bmj.c181.long.

Please remove or anonymize all personal information (<specific identifying information in file to be removed>), ensure that the data shared are in accordance with participant consent, and re-upload a fully anonymized data set. Please note that spreadsheet columns with personal information must be removed and not hidden as all hidden columns will appear in the published file.

4. If the reviewer comments include a recommendation to cite specific previously published works, please review and evaluate these publications to determine whether they are relevant and should be cited. There is no requirement to cite these works unless the editor has indicated otherwise.

Additional Editor Comments:

When responding to Reviewers' comments, the authors should also generally improve the methodology, including specifying the criteria used to diagnose samples used in the study, and also the sampling procedure. The authors should also highlight all the limitations to this study, such as when suggesting their novel test as confirmatory with only 40% sensitivity.

[Note: HTML markup is below. Please do not edit.]

Reviewers' comments:

Reviewer's Responses to Questions

**Comments to the Author**

1. Is the manuscript technically sound, and do the data support the conclusions?

Reviewer #1: Partly

Reviewer #2: Yes

**********

2. Has the statistical analysis been performed appropriately and rigorously?

Reviewer #1: Yes

Reviewer #2: No

**********

3. Have the authors made all data underlying the findings in their manuscript fully available?

The PLOS Data policy

Reviewer #1: Yes

Reviewer #2: Yes

**********

4. Is the manuscript presented in an intelligible fashion and written in standard English?

Reviewer #1: Yes

Reviewer #2: No

**********

Reviewer #1: ABSTRACT

The unstructured abstract contains sufficient, brief information about the article. I do not have an extra recommendation here.

INTRODUCTION

This section contains some abbreviations that are not fully explained when first mentioned in the text, such as CDO1. The authors should use the full forms first, then use the abbreviations. In addition, the authors clearly explain the study's objective.

METHODS

The strength of the study is that written informed consent was obtained from the patients, despite the study being retrospective. The authors should explain why they chose to evaluate the pathological samples, including at least 30% of tumor cells. Why this ratio? According to which guideline?

Apart from these, the methods section is comprehensive and thoroughly explains each step used in the research.

RESULTS

Nomenclature confusion: The authors should use a single nomenclature for the group without gastritis and H pylori. However, they label them “normal” in one table and “control” in another.

All tables should include an explanation of the abbreviations as a footnote at the bottom. For example, the CDO1 explanation is missing in Table 3.

Apart from these, the results section includes sufficient illustrative examples and robust statistical analysis methods for this research.

In Table 1, the spelling of Famale should be corrected to Female.

DISCUSSION

Although the central finding indicates that CDO1 gene promoter methylation levels are significantly higher in the GC and HPP groups, there is no significant difference in CDO1 positivity between the groups. This disconnect fundamentally challenges the functional impact of the observed hypermethylation. While the authors discuss the potential reasons for this in the discussion section, the lack of mRNA expression data remains a significant limitation. Without demonstrating that promoter methylation actually leads to transcriptional silencing of CDO1, the study's core argument is notably weakened.

The second major concern is that the ROC analysis for differentiating GC from HPP-chronic gastritis, the authors suggest an alternative cut-off value of 164%, which yields a sensitivity of 40% and a specificity of 94%. Proposing a biomarker test with only a 40% sensitivity for cancer detection is clinically problematic since it would result in a high rate of false negatives. The rationale for suggesting this threshold as a “confirmatory tool” needs stronger clinical justification, or the limitations of this test’s diagnostic value should be stated more explicitly.

Final comment

As a clinician in internal medicine, I would be pleased to have a new emerging option to detect gastric cancers at earlier stages, thereby prolonging patients' survival. This study clinically supports this idea; however, there are major limitations. There are substantial discrepancies between the molecular findings and protein expression that remain unresolved. Additionally, concerns regarding sample purity and the proposed clinical utility of the statistical cut-offs need to be addressed before the manuscript can be considered for publication. Therefore, I recommend a major revision for this article.

Reviewer #2: This manuscript investigates the association between aberrant methylation of CDO1 promoter and gastric carcinogenesis. CDO1 is a tumor suppressor gene that has been reported to be associated with various cancers including gastric cancer. The authors conducted a cross-sectional comparative study on gastric cancer patients and controls along with H. Pylori positive and H. Pylori negative chronic gastritis patients to assess the clinical relevance of CDO1 gene promoter methylation and to validate it as a non-invasive marker for early diagnosis of gastric cancer in H. Pylori positive-chronic gastritis patients.

This study reported that the CDO1 gene promoter methylation levels were significantly higher in the gastric cancer group and the H. Pylori positive-chronic gastritis patients, compared to the H. Pylori negative chronic gastritis and control groups. However, there was no significant difference between the GC group and the H. Pylori positive-chronic gastritis group.

Recommendations

1. In the introduction section, epidemiology of gastric cancer could’ve been added to highlight the disease’s significance

2. The methodology section of this study lacks specific information regarding the diagnostic criteria used to confirm H.Pylori status of the patients included in the study cohort.

3. The authors reported a significant association between H. Pylori infection and CDO1 promoter hypermethylation. However, the manuscript lacks any discussion of bacterial virulence factors, particularly CagA that have been reported to accelerate the recruitment of DNA methyltransferases (DNMTs) to tumor suppressor promoters.

4. In the results section, Table 3 corresponds to the comparison of methylation levels and IHC scores across various groups. The authors performed Kruskal Wallis test, however they also reported p values for individual group comparisons. But Kruskal Wallis test does not provide pairwise comparison, so it needs to be specified that which test was used to get these significance levels.

5. The authors reported that there was no significant correlation between CDO1 gene promoter methylation levels and CDO1 immunopositivity in the study groups. However, the statistical analysis to support this is missing (i.e; Spearman correlation or Pearson’s correlation).

6. The manuscript should be thoroughly checked and corrected for grammatical mistakes.

**********

what does this mean?). If published, this will include your full peer review and any attached files.

If you choose “no”, your identity will remain anonymous but your review may still be made public.

**Do you want your identity to be public for this peer review?**  For information about this choice, including consent withdrawal, please see our Privacy Policy

Reviewer #1: No

Reviewer #2: No

**********

[NOTE: If reviewer comments were submitted as an attachment file, they will be attached to this email and accessible via the submission site. Please log into your account, locate the manuscript record, and check for the action link "View Attachments". If this link does not appear, there are no attachment files.]

To ensure your figures meet our technical requirements, please review our figure guidelines: https://journals.plos.org/plosone/s/figures

You may also use PLOS’s free figure tool, NAAS, to help you prepare publication quality figures: https://journals.plos.org/plosone/s/figures#loc-tools-for-figure-preparation.

NAAS will assess whether your figures meet our technical requirements by comparing each figure against our figure specifications.

# Author comment: Author response to Decision Letter 1

**DOI:** 10.1371/journal.pone.0335832.r002

29 Apr 2026

Journal Requirements:

1. Please ensure that your manuscript meets PLOS ONE's style requirements, including those for file naming.

“Our article's compliance with PLOS ONE's style requirements, including file naming, has been reviewed.”

2. Please state what role the funders took in the study. If the funders had no role, please state: "The funders had no role in study design, data collection and analysis, decision to publish, or preparation of the manuscript." If this statement is not correct, you must amend it as needed. Please include this amended Role of Funder statement in your cover letter; we will change the online submission form on your behalf.

“Funding statements and their roles are added in the cover letter.”

3. We note that there is identifying data in the Supporting Information file “S3_File_Data_set.xlsx”. Due to the inclusion of these potentially identifying data, we have removed this file from your file inventory. Prior to sharing human research participant data, authors should consult with an ethics committee to ensure data are shared in accordance with participant consent and all applicable local laws. Please remove or anonymize all personal information (<specific identifying information in file to be removed>), ensure that the data shared are in accordance with participant consent, and re-upload a fully anonymized data set. Please note that spreadsheet columns with personal information must be removed and not hidden as all hidden columns will appear in the published file.

“S3_File_Data_set.xlsx corrected. Ages and genders of the participants are removed.”

4. If the reviewer comments include a recommendation to cite specific previously published works, please review and evaluate these publications to determine whether they are relevant and should be cited. There is no requirement to cite these works unless the editor has indicated otherwise.

“The reviewers did not recommend citing any specific previously published works.”

Additional Editor Comments:

When responding to Reviewers' comments, the authors should also generally improve the methodology, including specifying the criteria used to diagnose samples used in the study, and also the sampling procedure. The authors should also highlight all the limitations to this study, such as when suggesting their novel test as confirmatory with only 40% sensitivity.

Author's Response:

We thank the Editor for these valuable and constructive comments. In response, we have carefully revised the manuscript to improve methodological clarity and to more explicitly address the study limitations.

First, the Methods section has been revised to provide clearer details regarding the diagnostic criteria and sampling procedures. Specifically, we have clarified that H. pylori infection status was determined by histopathological evaluation of gastric biopsy specimens using modified Giemsa staining, and we have further elaborated on the patient selection and sample processing procedures.

Second, we have expanded the Discussion section to more explicitly acknowledge the limitations of the study. In particular, we now clearly state the lack of mRNA expression data, the relatively small sample size, the absence of long-term follow-up, and the potential impact of sample heterogeneity.

Finally, we have revised the interpretation of the ROC analysis to avoid overstatement of clinical applicability. The discussion of the alternative cut-off value with lower sensitivity has been substantially tempered, and it is now clearly stated that CDO1 methylation alone may not be sufficient as a standalone diagnostic or confirmatory tool and should be interpreted with caution.

We believe that these revisions have significantly improved the clarity, transparency, and clinical interpretation of the study.

Response to Reviewer_1

We believe that these revisions have strengthened the manuscript and improved the clarity and balance of our interpretations. We thank the reviewer again for these valuable suggestions, which have significantly contributed to improving the quality of our work.

Section for INTRODUCTION

Reviewer comment:

This section contains some abbreviations that are not fully explained when first mentioned in the text, such as CDO1. The authors should use the full forms first, then use the abbreviations. In addition, the authors clearly explain the study's objective.

Author's Response:

We would like to thank the reviewer for this careful reading and the positive feedback regarding the clarity of our study's objective.

We have addressed the concern regarding abbreviations throughout the Introduction. Specifically, the full name for CDO1 (Cysteine Dioxygenase Type 1) and other relevant terms have been provided upon their first mention. We have also performed a thorough check of the entire manuscript to ensure all abbreviations are consistently defined.

Changes in the Manuscript:

The full forms of abbreviations have been added in the Introduction section and the entire article.

Section for METHODS

Reviewer comment:

The strength of the study is that written informed consent was obtained from the patients, despite the study being retrospective. The authors should explain why they chose to evaluate the pathological samples, including at least 30% of tumor cells. Why this ratio? According to which guideline? Apart from these, the methods section is comprehensive and thoroughly explains each step used in the research.

Author's Response:

We thank the reviewer for this insightful and constructive comment. We agree that clarification of the tumor cellularity threshold is important for methodological transparency.

Tumor cellularity is a critical factor in molecular pathology, as the presence of stromal, inflammatory, or normal epithelial cells may dilute tumor-derived DNA and reduce the sensitivity of molecular assays. Therefore, assessment of tumor cell content by a pathologist prior to molecular testing is widely recommended to ensure that the analyzed nucleic acids adequately represent the tumor component [1,2].

International molecular pathology guidelines, including those from the College of American Pathologists (CAP), the International Association for the Study of Lung Cancer (IASLC), and the Association for Molecular Pathology (AMP), emphasize the importance of evaluating tumor cellularity before molecular analysis to avoid false-negative results due to insufficient tumor DNA. https://www.cap.org/protocols-and-guidelines/cap-guidelines/current-cap-guidelines/molecular-testing-guideline-for-the-selection-of-lung-cancer-patients-for-treatment-with-tyrosine-kinase-inhibitors?utm_source=chatgpt.co. [1]. However, these guidelines do not define a strict universal cut-off, but rather recommend ensuring sufficient tumor content for reliable detection.

In clinical molecular pathology practice, tumor cellularity thresholds in the range of approximately 20–30% are commonly adopted for PCR-based assays, including methylation analyses, to ensure reliable detection of tumor-specific molecular alterations although no universally accepted cut-off has been formally established [1–5]. Although PCR-based methods can theoretically detect low levels of altered DNA under controlled conditions, the reliability of detection in formalin-fixed paraffin-embedded (FFPE) clinical samples is strongly influenced by tumor purity, sample heterogeneity, and pre-analytical variables [3–5]. When tumor cellularity is low, the increased proportion of non-tumoral DNA may dilute tumor-specific signals and compromise analytical sensitivity.

Based on these widely accepted principles and previous literature, we included only tissue sections containing at least 30% tumor cells, as evaluated by an experienced pathologist. This threshold was selected as a conservative and commonly used criterion to minimize contamination by non-neoplastic cells and to ensure adequate tumor-derived DNA for the quantitative methylation-specific PCR analysis performed in this study.

References

1. Lindeman NI, Cagle PT, Aisner DL, Arcila ME, Beasley MB, Bernicker EH, Colasacco C, Dacic S, Hirsch FR, Kerr K, Kwiatkowski DJ, Ladanyi M, Nowak JA, Sholl L, Temple-Smolkin R, Solomon B, Souter LH, Thunnissen E, Tsao MS, Ventura CB, Wynes MW, Yatabe Y. Updated Molecular Testing Guideline for the Selection of Lung Cancer Patients for Treatment With Targeted Tyrosine Kinase Inhibitors: Guideline From the College of American Pathologists, the International Association for the Study of Lung Cancer, and the Association for Molecular Pathology. Arch Pathol Lab Med. 2018 Mar;142(3):321-346. doi: 10.5858/arpa.2017-0388-CP. Epub 2018 Jan 22. PMID: 29355391.

2. van Krieken JH, Normanno N, Blackhall F, Boone E, Botti G, Carneiro F, Celik I, Ciardiello F, Cree IA, Deans ZC, Edsjö A, Groenen PJ, Kamarainen O, Kreipe HH, Ligtenberg MJ, Marchetti A, Murray S, Opdam FJ, Patterson SD, Patton S, Pinto C, Rouleau E, Schuuring E, Sterck S, Taron M, Tejpar S, Timens W, Thunnissen E, van de Ven PM, Siebers AG, Dequeker E. Guideline on the requirements of external quality assessment programs in molecular pathology. Virchows Arch. 2013 Jan;462(1):27-37. doi: 10.1007/s00428-012-1354-4. Epub 2012 Dec 19. PMID: 2325035

3. Jennings LJ, Arcila ME, Corless C, Kamel-Reid S, Lubin IM, Pfeifer J, Temple-Smolkin RL, Voelkerding KV, Nikiforova MN. Guidelines for Validation of Next-Generation Sequencing-Based Oncology Panels: A Joint Consensus Recommendation of the Association for Molecular Pathology and College of American Pathologists. J Mol Diagn. 2017 May;19(3):341-365. doi: 10.1016/j.jmoldx.2017.01.011. Epub 2017 Mar 21. PMID: 28341590; PMCID: PMC6941185.

4. Schmitz, M., Wunsch, K., Hoyer, H. et al. Performance of a methylation specific real-time PCR assay as a triage test for HPV-positive women. Clin Epigenet 9, 118 (2017). https://doi.org/10.1186/s13148-017-0419-2

5. Roy-Chowdhuri S, Stewart J. Preanalytic Variables in Cytology: Lessons Learned From Next-Generation Sequencing-The MD Anderson Experience. Arch Pathol Lab Med. 2016 Nov;140(11):1191-1199. doi: 10.5858/arpa.2016-0117-RA. Epub 2016 Jun 22. PMID: 27333361.

To address the reviewer’s comment, we have now clarified this point in the METHODS section of the revised manuscript (Materials and Methods: Study design and clinical samples: Last paragraph ) as follows:

“Only tissue sections containing at least 30% tumor cells, as assessed by an experienced pathologist, were included in the analysis to ensure sufficient tumor DNA for reliable PCR-based methylation analysis, in line with commonly adopted practices in molecular pathology [1,5].”

Section for RESULTS

Reviewer comment:

Nomenclature confusion: The authors should use a single nomenclature for the group without gastritis and H pylori. However, they label them “normal” in one table and “control” in another.

All tables should include an explanation of the abbreviations as a footnote at the bottom. For example, the CDO1 explanation is missing in Table 3.

Apart from these, the results section includes sufficient illustrative examples and robust statistical analysis methods for this research.

In Table 1, the spelling of Famale should be corrected to Female.

Author's Response:

In response to the nomenclature inconsistency, we have revised all tables and the main text to ensure the use of a single, consistent term. The group without gastritis and H. pylori infection is now uniformly referred to as the “control” group throughout the manuscript.

We also agree that clear definition of abbreviations improves readability. Accordingly, we have added explanatory footnotes for all abbreviations in each table. Specifically, the abbreviation “CDO1” has now been defined in Table 3, and similar clarifications have been provided wherever needed.

In addition, the typographical error in Table 1 has been corrected, and “Famale” has been revised to “Female”.

These changes have been incorporated into the revised manuscript (Tables 1–3) to improve clarity, consistency, and overall presentation.

Section for DISCUSSION

Reviewer comment:

Although the central finding indicates that CDO1 gene promoter methylation levels are significantly higher in the GC and HPP groups, there is no significant difference in CDO1 positivity between the groups. This disconnect fundamentally challenges the functional impact of the observed hypermethylation. While the authors discuss the potential reasons for this in the discussion section, the lack of mRNA expression data remains a significant limitation. Without demonstrating that promoter methylation actually leads to transcriptional silencing of CDO1, the study's core argument is notably weakened.

The second major concern is that the ROC analysis for differentiating GC from HPP-chronic gastritis, the authors suggest an alternative cut-off value of 164%, which yields a sensitivity of 40% and a specificity of 94%. Proposing a biomarker test with only a 40% sensitivity for cancer detection is clinically problematic since it would result in a high rate of false negatives. The rationale for suggesting this threshold as a “confirmatory tool” needs stronger clinical justification, or the limitations of this test’s diagnostic value should be stated more explicitly.

Author's Response:

We sincerely thank the reviewer for this insightful and important comment.

First; we fully agree that demonstrating the functional consequences of promoter methylation at the mRNA level would strengthen the biological interpretation of our findings. In the present study, we observed significantly increased CDO1 promoter methylation levels in both gastric cancer (GC) and H. pylori-positive chronic gastritis groups; however, this was not paralleled by a significant difference in protein expression as assessed by immunohistochemistry. As correctly pointed out, this discrepancy limits the ability to directly infer transcriptional silencing based solely on methylation data.

Importantly, our study was primarily designed to evaluate the potential of CDO1 promoter methylation as a biomarker rather than to comprehensively investigate its functional consequences. In this context, the observed differences in methylation levels between groups remain informative. Nevertheless, we have now more explicitly acknowledged that the lack of mRNA expression data represents an important limitation of the study.

We also acknowledge that the relationship between promoter methylation and gene expression is complex and not always linear. Gene expression may be influenced by multiple regulatory mechanisms, including histone modifications, alternative promoter usage, post-transcriptional regulation, and protein stability (Bu S, et al; Rauluseviciute I, et al). Therefore, the absence of a direct correlation between methylation status and protein expression does not necessarily exclude a biological role for CDO1 methylation.

In the revised manuscript, we have strengthened the Discussion section by (i) clearly stating the absence of mRNA expression data as a limitation, (ii) further tempering the interpretation of the functional implications of methylation, and (iii) emphasizing the need for future studies integrating methylation, transcriptional, and functional analyses to clarify the biological significance of CDO1 promoter methylation (Discussion: 13 -18. paragraph ).

Second; We agree that a sensitivity of 40% is relatively low for cancer detection and may result in a substantial number of false-negative cases if used as a standalone diagnostic tool.

In the original version, the interpretation of this threshold may have been overly suggestive of clinical applicability. In response to the reviewer’s comment, we have substantially revised and tempered this interpretation in the Discussion section.

Specifically, we now emphasize that although different cut-off values illustrate the trade-off between sensitivity and specificity, the higher threshold (16.4) is associated with limited sensitivity and may result in a considerable number of false-negative cases.

Importantly, we have removed the previous implication that this threshold could be used as a “confirmatory tool” and instead clearly state that CDO1 methylation alone is insufficient as a standalone diagnostic approach, particularly in the clinically

Attachment Submitted filename: Response to Reviewers.docx

# Peer review: Decision Letter 1

**DOI:** 10.1371/journal.pone.0335832.r003

**Authors:** Elingarami Sauli

29 Jul 2026

A comparative study of CDO1 promoter methylation in gastric cancer and H. pylori-associated chronic gastritis: Implications for diagnostic performance

PONE-D-25-51757R1

Dear Dr. Selin,

We’re pleased to inform you that your manuscript has been judged scientifically suitable for publication and will be formally accepted for publication once it meets all outstanding technical requirements.

Within one week, you’ll receive an e-mail detailing the required amendments. When these have been addressed, you’ll receive a formal acceptance letter and your manuscript will be scheduled for publication.

An invoice will be generated when your article is formally accepted. Please note, if your institution has a publishing partnership with PLOS and your article meets the relevant criteria, all or part of your publication costs will be covered. Please make sure your user information is up-to-date by logging into Editorial Manager at Editorial Manager® and clicking the ‘Update My Information' link at the top of the page. For questions related to billing, please contact billing support.

If your institution or institutions have a press office, please notify them about your upcoming paper to help maximize its impact. If they’ll be preparing press materials, please inform our press team as soon as possible -- no later than 48 hours after receiving the formal acceptance. Your manuscript will remain under strict press embargo until 2 pm Eastern Time on the date of publication. For more information, please contact onepress@plos.org.

Kind regards,

Elingarami Sauli, PhD

Academic Editor

PLOS One

Additional Editor Comments (optional):

To this end....the reviewers have satisfactorily responded to all originally given comments....hence the submission can be accepted.

Staff Editor Comments:

PLOS One is an inclusive journal that welcomes all rigorous science that contributes to the base of academic knowledge (https://journals.plos.org/plosone/s/criteria-for-publication#loc-1). As stated in our journal information page, manuscripts are assessed on the basis of methodological rigor and high ethical standards, regardless of perceived novelty (https://journals.plos.org/plosone/s/journal-information).

Reviewers' comments:

Reviewer's Responses to Questions

**Comments to the Author**

Reviewer #3: All comments have been addressed

Reviewer #4: All comments have been addressed

**********

2. Is the manuscript technically sound, and do the data support the conclusions?

Reviewer #3: Yes

Reviewer #4: No

**********

3. Has the statistical analysis been performed appropriately and rigorously?

Reviewer #3: Yes

Reviewer #4: No

**********

4. Have the authors made all data underlying the findings in their manuscript fully available?

The PLOS Data policy

Reviewer #3: Yes

Reviewer #4: Yes

**********

5. Is the manuscript presented in an intelligible fashion and written in standard English?

Reviewer #3: Yes

Reviewer #4: No

**********

Reviewer #3: The authors have satisfactorily addressed the reviewers' comments and have significantly improved the manuscript. The revisions have enhanced the methodological transparency, statistical reporting, and discussion of limitations. I have no further major concerns and consider the manuscript suitable for publication in its current form.

Reviewer #4: the manuscript are well written but...

This manuscript lacks good innovation and, in my opinion, is not suitable for publication.

**********

what does this mean?). If published, this will include your full peer review and any attached files.

If you choose “no”, your identity will remain anonymous but your review may still be made public.

**Do you want your identity to be public for this peer review?**  For information about this choice, including consent withdrawal, please see our Privacy Policy

Reviewer #3: **Yes:**  Sinem Oktem Okullu

Reviewer #4: No

**********

# Editor report: Acceptance letter

**DOI:** 10.1371/journal.pone.0335832.r004

**Authors:** Elingarami Sauli

PONE-D-25-51757R1

PLOS One

Dear Dr. Kankaya,

I'm pleased to inform you that your manuscript has been deemed suitable for publication in PLOS One. Congratulations! Your manuscript is now being handed over to our production team.

At this stage, our production department will prepare your paper for publication. This includes ensuring the following:

* All references, tables, and figures are properly cited

* All relevant supporting information is included in the manuscript submission,

* There are no issues that prevent the paper from being properly typeset

You will receive further instructions from the production team, including instructions on how to review your proof when it is ready. Please keep in mind that we are working through a large volume of accepted articles, so please give us a few days to review your paper and let you know the next and final steps.

Lastly, if your institution or institutions have a press office, please let them know about your upcoming paper now to help maximize its impact. If they'll be preparing press materials, please inform our press team within the next 48 hours. Your manuscript will remain under strict press embargo until 2 pm Eastern Time on the date of publication. For more information, please contact onepress@plos.org.

You will receive an invoice from PLOS for your publication fee after your manuscript has reached the completed accept phase. If you receive an email requesting payment before acceptance or for any other service, this may be a phishing scheme. Learn how to identify phishing emails and protect your accounts at https://explore.plos.org/phishing.

If we can help with anything else, please email us at customercare@plos.org.

Thank you for submitting your work to PLOS ONE and supporting open access.

Kind regards,

PLOS ONE Editorial Office Staff

on behalf of

Dr. Elingarami Sauli

Academic Editor

PLOS One
